# TRIM25 degrades BRD7 protein stability through the ubiquitin proteasome pathway to promote breast cancer progression and paclitaxel resistance by activating YB1/Bcl-2 transcription axis

**DOI:** 10.1038/s41419-025-08140-8

**Published:** 2025-11-28

**Authors:** Changning Xue, Qingqing Wei, Mengna Li, Jianxia Wei, Lemei Zheng, Zubing Wu, Huizhen Xin, Yumei Duan, Songqing Fan, Wei Xiong, Guiyuan Li, Faqing Tang, Hongyu Deng, Ming Zhou

**Affiliations:** 1 NHC Key Laboratory of Carcinogenesis, Hunan Key Laboratory of Oncotarget Gene, Hunan Cancer Hospital and the Affiliated Cancer Hospital of Xiangya School of Medicine, Central South University, Changsha, China; 2 Cancer Research Institute and School of Basic Medical Sciences, Central South University, Changsha, China; 3 The Key Laboratory of Carcinogenesis and Cancer Invasion of the Chinese Ministry of Education, Central South University, Changsha, China; 4 Department of Pathology, the Second Xiangya Hospital, Central South University, Changsha, China

**Keywords:** Breast cancer, Ubiquitylation, Apoptosis

## Abstract

Paclitaxel (PTX) is one of the most common chemotherapeutic drugs for treating breast cancer (BC), but resistance to PTX chemotherapy remains the major cause of treatment failure in BC patients. Our previous studies demonstrated that BRD7 participates in the paclitaxel-mediated chemotherapy sensitization and inhibits the malignant progression of breast cancer. Furthermore, TRIM25 was screened by IP-MS as a potential E3 ubiquitin ligase interacting with BRD7. Nevertheless, the functions and mechanisms of TRIM25 in the malignant progression of breast cancer and PTX resistance, as well as its regulatory relationship with BRD7, are still not clear. Our investigation revealed that TRIM25 effectively promoted cell proliferation, cell cycle progression, and paclitaxel chemoresistance of BC cells. Mechanistically, TRIM25 interacted with BRD7, and the PRYSPRY region of TRIM25 bond to the N-terminal region of BRD7. Additionally, TRIM25 decreased the protein stability of BRD7 through the ubiquitin proteasome pathway by increasing the K48-linked ubiquitination of BRD7 at the K119 site, and then activated the YB1/Bcl-2 signal axis, thus mediating malignant progression and PTX resistance of breast cancer. We further demonstrated that restoration of BRD7 rescued the inhibitory effect of TRIM25 knockdown on the malignant progression and PTX resistance of BC cells. Furthermore, high expression of TRIM25 was found in clinical breast cancer tissues compared to noncancerous breast tissues, which was positively associated with poor prognosis in BC patients. The expression of TRIM25 was negatively correlated with BRD7 expression, and the combined expression of TRIM25 and BRD7 might be a potential molecular marker for the prediction of malignant progression and prognosis of breast cancer. Our findings demonstrate that targeting the TRIM25/BRD7/YB1/Bcl-2 signal axis might be a potential therapeutic strategy for the treatment of breast cancer.

## Introduction

In recent years, the incidence of breast cancer in the world continues to increase; it accounts for 31% of the confirmed cases of female cancer according to the statistics of GLOBOCAN in 2023 [1]. Chemotherapy has played an important role in the comprehensive treatment of breast cancer, especially the recurrent and metastatic breast cancer [2]. Paclitaxel (PTX) has been widely used in clinical breast cancer chemotherapy [3, 4], whereas PTX resistance of breast cancer is closely related to recurrence and distant metastasis of breast cancer patients, which is a major obstacle and difficulty in the treatment of breast cancer [5]. Therefore, it is of great clinical significance to clarify the molecular mechanism of occurrence and development and PTX resistance of breast cancer for exploring more effective therapeutic targets and improving the prognosis of breast cancer patients.

Bromodomain-containing protein 7 (BRD7) is a protein containing a single bromodomain (Brd), and it is a member of the SWI/SNF chromatin remodeling complex [6]. BRD7 has been reported to be involved in breast cancer proliferation, invasion, metastasis, and PTX resistance [7–9]. Our previous study reported that BRD7 promotes PTX-mediated breast cancer cell apoptosis and enhances the chemosensitivity of PTX by upregulating Bak expression at the transcription level [7]. The suppressive role of BRD7 in cancers has been well recognized, whereas the mechanism of BRD7 in PTX resistance in breast cancer remains unclear. It has been demonstrated that sumoylation, phosphorylation, and ubiquitination are involved in the post-translational modification of BRD7 protein [10–12], suggesting that the post-translational modification plays an important role in the regulation of BRD7 expression. To further reveal the underlying mechanism of PTX resistance, we used IP-MS analysis to search for proteins interacting with BRD7 in BC cells and found TRIM25 interacting with BRD7. However, the functions and mechanisms of TRIM25 in the malignant progression and PTX resistance of breast cancer, as well as the regulatory relationship between TRIM25 and BRD7, have not been determined.

Tripartite motif protein 25 (TRIM25), containing a RING domain, one or two B-box motifs, a Coiled-Coil domain, and a PRYSPRY domain [13], in which the RING domain endows TRIM25 with E3 ubiquitin ligase activity. TRIM25, a potential E3 ubiquitin ligase, promotes the ubiquitination and degradation of its substrates, playing vital roles in tumor cell proliferation, apoptosis, immune regulation, and antiviral responses [13–15]. Accumulating evidence demonstrates that TRIM25 is a key player in tumor chemotherapy resistance [16, 17]. TRIM25 drives chemotherapy resistance by regulating autophagy and modifying splicing factors [18, 19]. For example, studies demonstrated that TRIM25 stabilizes EZH2, promoting oxaliplatin resistance in colorectal cancer cells [20]. In triple-negative breast cancer (TNBC), neddylated TRIM25 facilitates K63-linked ubiquitination and nuclear translocation of TFEB (transcription factor EB), activating autophagy-related genes and inducing PTX resistance [18]. However, the precise functions and molecular mechanisms of TRIM25 in breast cancer pathogenesis and PTX chemotherapy resistance remain to be fully elucidated.

Y-box binding protein (YB1) belongs to the Y Box family, which contains both a cold shock domain (CSD) and a Y-box domain, and is a DNA/RNA binding protein [21]. Studies have shown that YB1 is highly expressed in breast cancer, and this expression correlates with tumor malignant progression and poor overall survival [22, 23]. In our previous study, BRD7 interacted with YB1 and negatively regulated the phosphorylation of YB1 at Ser102, promoting the ubiquitination and degradation of YB1, thus effectively inhibiting the invasion and metastasis of breast cancer cells [8]. Bcl-2 family members contain conserved domains such as BH1, BH2, BH3, and BH4, which have high homology and play a vital role in the process of apoptosis [24]. Bcl-2 is an important anti-apoptosis molecule in the Bcl-2 family. Various studies revealed that Bcl-2 played a critical role in apoptosis and the anti-tumor pathway mediated by PTX, and the low expression of Bcl-2 was the key to apoptosis and the anti-tumor effect mediated by PTX [25–27]. We found that YB1 could potentially bind to the promoter region of Bcl-2 and increase its expression at the mRNA level. Therefore, BRD7 may inhibit the malignant progression of breast cancer and improve the chemotherapy sensitivity of PTX by negatively regulating the activity of the YB1/Bcl-2 transcription axis, which needs further investigation.

Herein, we reported that TRIM25 directly interacted with BRD7, and promoted the degradation of BRD7 through the ubiquitin-proteasome mediated pathway in breast cancer cells as an E3 ligase, and then activated YB1/Bcl-2 transcription axis, thus promoting the malignant progression and chemotherapy resistance of PTX of breast cancer in vitro and in vivo. Moreover, we further validated that high TRIM25 expression predicts poor prognosis of BC patients, and a combination of TRIM25 and BRD7 expression is an effective marker for the prognosis of breast cancer. Altogether, this study verified the mechanism by which the TRIM25/BRD7/YB1/Bcl-2 axis is involved in the malignant progression and PTX resistance of breast cancer, providing a novel insight into BC development and potential targets for improving the PTX therapeutic resistance.

## Materials and methods

### Cells and cell culture

MDA-MB-231 and MCF7 cells were purchased from the American Type Culture Collection (ATCC), and the paclitaxel-resistant MDA-MB-231 cell line (MDA-MB-231-PR) was gifted from Professor Shujuan Fu at Hunan Normal University School of Medicine and all of these cells were cultured in DMEM (C3103-0500, Vivacell, Shanghai, China) supplemented with 10% fetal bovine serum (Vivacell, C04001-500) and 1% penicillin/streptomycin (NCM, C100C5). The cells were maintained at 37 °C in an incubator with 5% CO_2_.

### Plasmids and cell transfection

The pIRES2-EGFP-Flag-TRIM25, pCMV-HA-TRIM25, pCMV-HA-BRD7, pCMV-HA-YB1, pCMV-HA-Ub, pCMV-HA-K48-Ub, and pCMV-HA-K63-Ub plasmids were preserved in our laboratory. The BRD7 and TRIM25 deletion mutants plasmids and the putative ubiquitination sites mutations of BRD7 (K21R, K28R, K52R, K103R, K119R, and K127R) were constructed by us. The specific small interfering RNAs (siRNAs) targeting TRIM25 or BRD7 were purchased from RiboBio (Guangzhou, China). Plasmids and siRNAs were transfected into cells using Polyplus transfection (INTERFERin) according to the manufacturer’s protocol. The sequences of BRD7 siRNA wad described in our previous publication [28]. The sequences of the TRIM25 siRNAs were as follows: siTRIM25#1: 5′-GGGTCAACAGCAAGTTTGA-3′; siTRIM25#2: 5′-GCACCATAGACCTCAAAAA-3′.

### Proliferation and colony formation assays

For the proliferation assay, the cell viability of breast cancer cells was quantified by Cell Counting Kit-8 (CCK-8, B34304, Selleck, Houston, TX, USA). Briefly, 1000 cells/100 μl of DMEM medium were plated in each well of a 96-well plate. At each time point, 10 μL CCK-8 was added to each well, and after 2 h of incubation at 37 °C, the absorbance at 450 nm was recorded using a microplate reader (Beckman, Brea, CA, USA). For the colony formation assay, 1 × 10^3^ cells were planted in a 6-well plate and cultured at 37 °C. Finally, the cells were then fixed with 4% polyoxymethylene (G1101, Servicebio, Wuhan, China) for 30 min and stained with 0.1% crystal violet staining solution (C0121, Beyotime). The colonies were counted and normalized to the control.

### Western blot analysis

Cells were harvested and lysed in RIPA buffer (WB 3100, NCM Biotech, Suzhou, China). Total cell protein was extracted, and the protein concentration was determined with the BCA protein assay kit (WB 6501, NCM Biotech, Suzhou, China). Total proteins were separated on 10% SDS-PAGE and then transferred onto PVDF membranes (Millipore), blocked with 5% nonfat milk. The membranes were incubated with the indicated primary antibodies at 4 °C overnight. The primary antibodies used were anti-BRD7 (Cat No. 51009-2-AP, Proteintech, Wuhan, China), anti-TRIM25 (Cat No. 67314-1-Ig, Proteintech, Wuhan, China), anti-Ub (Cat No. sc-9133, Santa Cruz, USA), anti-GAPDH (Cat No.10494-1-AP, Proteintech, Wuhan, China), anti-Flag (Cat No. 20543-1-AP, Proteintech, Wuhan, China), anti-HA (Cat No. M180-11 MBL, Beijing, China), anti-CDK4 (Cat No. 11026-1-AP, Proteintech, Wuhan, China), anti-CDK1 (Cat No.CY5177, Abways, Shanghai, China), anti-p21 (Cat No.2947, CST, MA, USA), anti-cleaved-PARP (Cat No.AF7023, Affinity, Jiangsu, China), anti-YB1 (Cat No. CY5462, Abways, Shanghai, China), and anti-Bcl-2 (Cat No. 80313-1-RR, Proteintech, Wuhan, China). The membranes were incubated with HRP-conjugated secondary antibodies for 1 h at 37 °C, and the signals were visualized using an ECL kit (BMU 102, Abbkine, Wuhan, China).

### Total RNA extraction, reverse transcription PCR, and qPCR

Total RNAs were isolated using TRIzol reagent (Sangon Biotech). cDNAs were prepared using HiScript III 1st Strand cDNA Synthesis Kit (+gDNA wiper) (Vazyme) according to the manufacturer’s protocol. The qRT-PCR analysis was performed by the SYBR green method (YEASEN). The sequences of the PCR primers for the corresponding human gene were provided in Table 1.

**Table 1:** The RT-qPCR primer sequences used for the detection of TRIM25 and BRD7.

Name	Primer sequences (5′-3′)	Primer Length (bp)
BRD7 Forward	AAGCACACGCCTTCAAGAGT	20
BRD7 Reverse	TTCCTTCACGATGCGGTCAA	20
TRIM25 Forward	AGAGCCTGACCAAGAGGGAT	20
TRIM25 Reverse	GTGGATTTGTGTGTGGACGC	20
GAPDH Forward	CAACGGATTTGGTCGTATTGG	21
GAPDH Reverse	TGACGGTGCCATGGAATTT	19

### Immunofluorescence

For immunofluorescence (IF), the breast cancer cells were cultured on glass coverslips in a 12-well plate. The cells were washed with PBS and fixed with 4% polyoxymethylene (G1101, Servicebio, Wuhan, China) for 30 min and then permeabilized with 0.3% Triton X-100 (DH351–5, Genview, China) for 30 min at room temperature, and blocked with normal goat serum (AR0009, BOSTER Biological Technology) for 1 h. Then the BC cells were incubated with the primary antibodies (anti-TRIM25, 1:400, and anti-BRD7, 1:400) overnight at 4 °C. Next, the cells were washed three times with PBS and incubated with secondary antibodies (1:1000, Thermo Fisher Scientific) for 1 h at room temperature, and nuclei were stained with DAPI (Beyotime Biotechnology, China) for 10 min at room temperature. A confocal laser scanning microscope was used to capture fluorescence images, and ImageJ was used to quantitatively analyze the co-localization of TRIM25 and BRD7 in BC cells [29, 30].

### Co-immunoprecipitation

For co-immunoprecipitation (Co-IP), BC cells were harvested and lysed in Western and IP lysis buffer (P70100, NCM Biotech, Suzhou, China) containing protease inhibitors after 48 h of transfection. The protein A/G-magnetic beads (B23201, Selleck, Houston, TX, USA) were incubated with the indicated antibodies (anti-Flag or anti-HA) for 2 h at room temperature. The cell lysates were incubated with prepared Protein A/G-magnetic beads overnight at 4 °C. The beads were washed with Western and IP lysis buffer, resuspended in SDS loading buffer, boiled, and detected by Western blotting with appropriate antibodies.

### Ubiquitination analysis

For BRD7 ubiquitination detection, BC cells were transfected with the indicated plasmids. 48 hours after transfection, cells were treated with the proteasome inhibitor MG132 (20 µM) for 4 h and lysed with Western and IP lysis buffer (P70100, NCM Biotech, Suzhou, China) containing cocktail. The protein A/G-magnetic beads (B23201, Selleck, Houston, TX, USA) were incubated with the BRD7 antibodies for 2 h at room temperature. The cell lysates were incubated with prepared Protein A/G-magnetic beads overnight for 12~14 h at 4 °C. The beads were then washed with Western and IP lysis buffer and boiled in 1 × SDS loading buffer for 7 min. The co-precipitates were analyzed by Western blotting analysis.

### Protein stability analysis

For the BRD7 protein stability detection, BC cells were transfected with the indicated plasmids for 24 h and incubated with cycloheximide (CHX) (50 μM, MCE, New Jersey, USA) at each time point (0, 1, 2, and 4 h). Cells were harvested and lysed in RIPA buffer (WB 3100, NCM Biotech, Suzhou, China) with cocktail, and the BRD7 protein expression was detected by western blotting analysis.

### Cell cycle analysis

Briefly, MDA-MB-231, MCF7, and PTX-resistant BC cells were seeded in a 6-well plate. After cells were transfected with the indicated plasmids for 24 h, cells were digested by trypsin and diluted with PBS. And then, 5 μL of PI was added and protected from light at 37 ◦C for 20 min. The cell cycle was detected by flow cytometry (Beckman Coulter, USA).

### Apoptosis assay

For the cell apoptosis assay, BC cells and PTX-resistant BC cells were planted into a 6-well plate, and treated with paclitaxel (PTX, HY-B0015, MCE, New Jersey, USA) for 48 h after transfection. Cell apoptosis rate was analyzed by flow cytometry using an Annexin V-fluorescein isothiocyanate(FITC)/propidium iodide (PI) apoptosis detection kit (BD Biosciences). In brief, BC cells were collected and washed twice with cold PBS. The cells were resuspended in 1×binding buffer containing Annexin V-FITC (5 μL) and PI (5 μL) in the dark for 20 minutes at room temperature. The cell samples were detected with a flow cytometer (DxP Athena), and the results were analyzed with FlowJo V10 software.

### Xenograft tumor model

Four-week-old female BALB/c nude mice were purchased from Hunan Slake Jingda Experimental Animal Co., Ltd. All mice were housed in the Laboratory Animal Center of Central South University. For the xenograft tumor model, the nude mice were randomly divided into six groups (*n* = 5 per group): MCF7/siNC, MCF7/siTRIM25, MCF7/siTRIM25 plus siBRD7, MCF7/siNC plus PTX, MCF7/siTRIM25 plus PTX, MCF7/siTRIM25 plus siBRD7 plus PTX. The indicated MCF7 cells were resuspended in 150 µL NaCl which contains 4 × 10^6^ cells. Then the cells were implanted into the dorsal flank of each mouse. For tumors treated with PTX, mice received 15 mg/kg PTX every two days during the experiment, four times in total. From the sixth day, the mice were treated with the negative control siRNA, siTRIM25, or siTRIM25 combined with siBRD7 (5 nmol/20 g, RiboBio, Guangzhou, China) every 4 days through intratumor injection. Tumor volumes were calculated every 2 days after at the sixth day, and the volume calculation formula was (length×width^2^)/2. All mice were sacrificed 22 days after implantation, and the tumors were harvested, weighed, and embedded in 4% formalin for IHC staining.

### ChIP-qPCR

ChIP assays were performed using 10 million MCF7 cells. ChIP was described in our previous work [31]. Briefly, MCF7 cells were crosslinked with 1% formaldehyde and then harvested and lysed. Nuclei lysis buffer was added to the precipitate, and the chromatin was broken up into fragments of 100–500 bp by a BioRuptor sonicator. Chromatin was immunoprecipitated with immunoglobulin IgG (Sigma) and anti-YB1 antibody (Abways) with the Protein A/G Magnetic Beads system (B23201, Selleck, Houston, TX, USA) according to the manufacturer’s protocols. Finally, the collected DNA was used for subsequent RT-qPCR. The primer sequences used for the ChIP-qPCR assay are listed in Table 2.

**Table 2:** The primer sequences for the ChIP-qPCR assay.

Name	Sequences (5′-3′)	Primer Length (bp)
ChlP-P1-Forward	GGTGCTTTGTCAAAGACTCTTGG	23
ChlP-P1- Reverse	ACCTTTGCCTCGTAGCCAAT	20
ChlP-P2-Forward	ATTGGCTACGAGGCAAAGGT	20
ChlP-P2- Reverse	AGGCCATGCAGAACTCAGC	19
ChlP-P3-Forward	GTGTGTAGGCGCGTGT	16
ChlP-P3- Reverse	TCCCGGGGAACCGCACG	17
ChlP-P4-Forward	CCCGGGAGCCCCCA	14
ChlP-P4- Reverse	ATGGCGCGCGGGGCC	15
ChlP-P5-Forward	CATGTGCCCCCGGCG	15
ChlP-P5- Reverse	AGCGGGGCTGTGGTG	15

### Immunohistochemical staining

Immunohistochemical staining (IHC) was performed as previously described by the research group [12]. At least three random fields per sample were examined. Sections were incubated with anti-BRD7 (Proteintech, Wuhan, China), anti-TRIM25 (Proteintech, Wuhan, China), anti-YB1 (Abways, Shanghai, China), anti-Bcl-2 (Proteintech, Wuhan, China), anti-p21 (CST, MA, USA), anti-CDK4 (Proteintech, Wuhan, China), anti-CDK1 (Abways, Shanghai, China), anti-cleaved-PARP (Affinity, Jiangsu, China), and anti-Ki67 (Bioworld, USA). MaxVisionTM HRP-Polymer anti-Mouse/Rabbit was incubated at room temperature for 30 min. After DAB staining, the nuclei were counterstained with hematoxylin (Solarbio, Beijing, China). The IHC score was calculated based on the staining intensity and the percentage of stained cells [7]. The final score is the product of the intensity score and the positive rate score. The expression level was classified as low or high with the median total score as the cutoff.

### Clinical data analysis

A total of 219 breast cancer and 34 normal breast paraffin-embedded samples were collected from the Second Xiangya Hospital of Central South University, and this study was approved by the Ethics Review Committees/Institutional Review Boards of Central South University. The clinicopathologic features of the breast cancer patients mainly included gender, age, tumor size, node metastasis, distant metastasis, clinical tumor node metastasis (TNM) stage, pathology diagnosis, survival time, and molecular subtype. The immunohistochemical scores of clinical samples were based on the detailed procedures described in our previous articles [7, 32].

### Statistical analysis

All data were statistically analyzed using GraphPad Prism (version 8.0.2, GraphPad Software, CA, USA). The data are presented as the mean ± standard deviation (SD) values as indicated in the figure legends. Two-tailed unpaired Student’s *t*-test was used to compare two groups of data. Two-tailed paired Student’s *t*-test was used to compare data for matched BC tissues. The chi-square test was used for comparison of categorical data, and Spearman correlation analysis was used for comparison of continuous variables. Survival curves were generated using Kaplan-Meier estimates, and differences between the survival curves were compared using the log-rank test. *P* values < 0.05 were considered to be statistically significant.

## Results

### TRIM25 promotes cell proliferation and cell cycle progression of BC cells

**Fig. 1:**
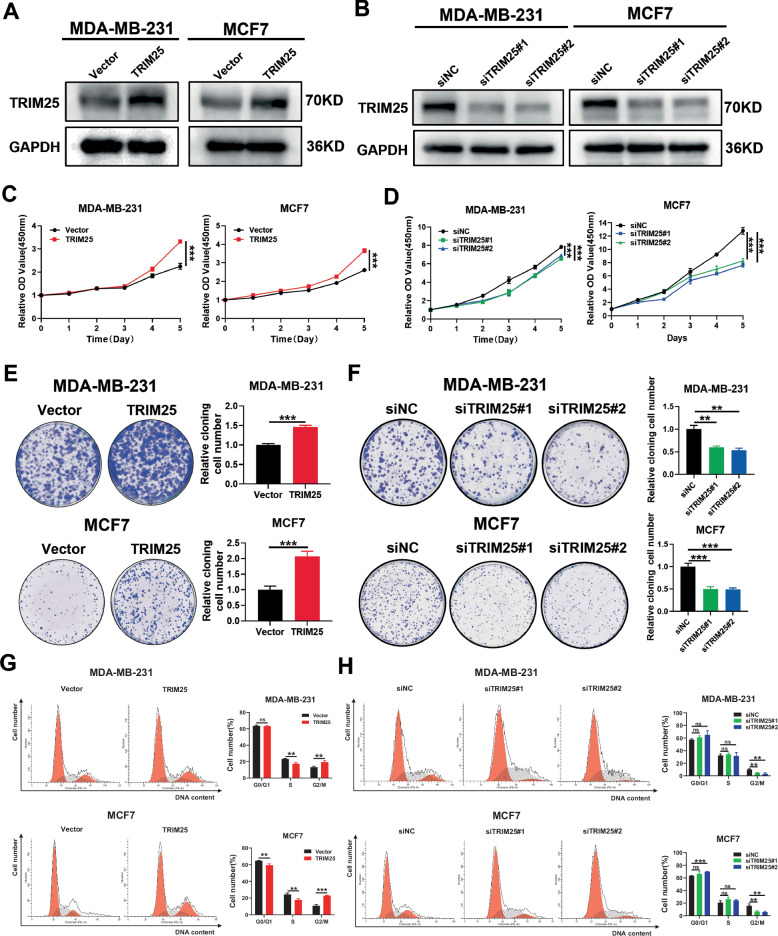
TRIM25 promotes cell proliferation and cell cycle progression of BC cells. **A** Effective overexpression of TRIM25 in MDA-MB-231 and MCF7 cells was verified by western blot. **B** Western blot analysis of TRIM25 protein level in MDA-MB-231 and MCF7 cells transfected with siNC, siTRIM25#1 or siTRIM25#2. **C** TRIM25 overexpression promoted cell proliferation detected by CCK-8 assay in BC cells. **D** MDA-MB-231 and MCF7 cells transfected with siNC, siTRIM25#1 or siTRIM25#2 were tested for cell viability using the CCK-8 assay. **E** Colony forming assay was performed in MDA-MB-231 and MCF7 cells transfected with Vector or TRIM25. **F** Colony formation assay on MDA-MB-231 and MCF7 cells after TRIM25 knockdown. **G** Cell-cycle analysis by flow cytometry in MDA-MB-231 and MCF7 cells with TRIM25 overexpression. **H** Cell-cycle analysis by flow cytometry in MDA-MB-231 and MCF7 cells with TRIM25 knockdown. Data are shown as the mean ± SD of at least three independent experiments, and the significant level was identified by ns, no significance, **P < 0.01, and ***P < 0.001.

The dysregulation of BRD7 expression in breast cancer is an important mechanism leading to tumor occurrence, development, and PTX resistance. Our previous study showed that BRD7 was an unstable protein in breast cancer, and that the ubiquitin-proteasome pathway was involved in the regulation of BRD7 protein instability [12]. To further reveal the mechanism of BRD7’s involvement in development and PTX chemotherapy sensitization in breast cancer, we screened and identified a potential E3 ubiquitin ligase, TRIM25, interacting with BRD7 by IP-MS in our previous study [12]. In order to determine the biological functions of TRIM25, we first constructed TRIM25-overexpressing and TRIM25 knockdown cells using MDA-MB-231 and MCF7 cell lines, the efficiency of which was confirmed by western blot assay (Fig. 1A, B). The Cell Counting Kit-8 (CCK-8) assay was performed to explore the effect of TRIM25 on the proliferation of breast cancer cells. The results showed that the overexpression of TRIM25 significantly promoted the proliferation of BC cells, while the knockdown of TRIM25 reduced the proliferation ability of BC cells (Fig. 1C, D). Furthermore, the colony formation assay showed that knockdown of TRIM25 decreased colony formation capacity, while the overexpression of TRIM25 led to the opposite result (Fig. 1E, F). Next, we analyzed the effect of TRIM25 on the cell cycle. As shown in Fig. 1G, H, the overexpression of TRIM25 resulted in an increased percentage of BC cells in the G2/M phase compared with the control, while the inhibition of TRIM25 decreased the percentage of cells in the G2/M phase in breast cancer cells. These results indicate that TRIM25 facilitates the cell cycle progression and proliferation of BC cells, suggesting a tumor-promoting role of TRIM25 in breast cancer.

### TRIM25 promotes chemoresistance of BC cells to paclitaxel

**Fig. 2:**
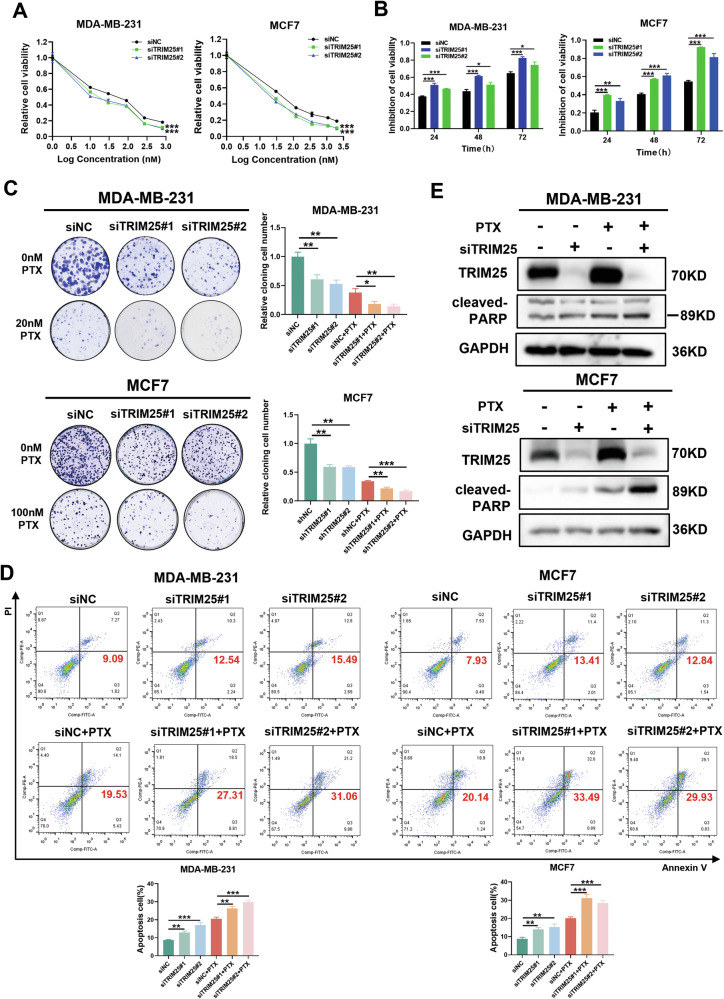
TRIM25 knockdown promotes chemosensitivity of BC cells to paclitaxel. **A** The TRIM25 knockdown BC cells were treated with PTX (MDA-MB-231: 0 nM, 1 nM, 10 nM, 30 nM, 90 nM, 270 nM, 810 nM, MCF7: 0 nM, 1 nM, 30 nM, 120 nM, 360 nM, 1080 nM, 2000 nM), cell viability was detected by CCK-8 assay. **B** MDA-MB-231 cells were treated with 20 nM PTX, and MCF7 cells were treated with 100 nM PTX, CCK-8 assay was performed to detect the inhibition rate of BC cells after TRIM25 knockdown at different time points. **C** The indicated BC cells were treated with paclitaxel (MDA-MB-231: 0 nM and 20 nM, MCF7: 0 nM and 100 nM) for 24 h, and colony formation ability was evaluated by a colony formation assay. **D** Annexin V-FITC and PI staining showing apoptosis in the TRIM25 knockdown BC cells treated with or without PTX (MDA-MB-231: 20 nM, MCF7: 100 nM) for 48 h. **E** MDA-MB-231 and MCF7 cells were treated with or without PTX (MDA-MB-231: 20 nM, MCF7: 100 nM) for 24 h, cleaved-PARP protein levels in BC cells with silent expression of TRIM25 were determined by western blot analysis. Data are shown as the mean ± SD of at least three independent experiments, and the significant level was identified by *P < 0.05, **P < 0.01, and ***P < 0.001.

We then investigated the role of TRIM25 in the process of paclitaxel resistance in BC cells. The TRIM25-knockdown or TRIM25-overexpressing BC cells were treated with different concentrations of PTX for 48 h, or treated with the same concentration of PTX for different times. The CCK-8 assay was used to evaluate cell viability. The downregulation of TRIM25 increased the sensitivity of BC cells to PTX (Fig. 2A, B), while the upregulation of TRIM25 decreased the sensitivity of cells to PTX (Fig. S1A, B). Subsequent colony formation assays also confirmed that the downregulation of TRIM25 enhanced the inhibitory effect of PTX on the colony-formation ability of BC cells (Fig. 2C), while overexpression of TRIM25 had the opposite result (Fig. S1C). We also performed cell apoptosis analysis, which showed that TRIM25 knockdown significantly increased PTX-induced apoptosis of BC cells (Fig. 2D), whereas overexpression of TRIM25 reduced PTX-induced apoptosis (Fig. S1D). Western blot analysis showed that TRIM25 knockdown promoted the expression of cleaved-PARP, while the expression of cleaved-PARP was decreased in the TRIM25 overexpression group. Meanwhile, PTX treatment enhanced the protein expression of cleaved-PARP compared was enhanced compared to that in untreated groups (Figs. 2E, S1E).

In order to further substantiate that TRIM25 was indeed related to paclitaxel resistance in breast cancer cells, a paclitaxel-resistant MDA-MB-231 cell line (MDA-MB-231-PR) was used for the following study. According to the result shown in Fig. S2A, compared with the parental MDA-MB-231 cells, PTX-resistant cells had a significantly higher IC50 value after a 48-h exposure to paclitaxel. The IC50 values of paclitaxel in the parental cell lines and resistant cell lines were 52.84 nM and 582.2 nM, respectively. We subsequently established TRIM25 overexpressed and knockdown paclitaxel-resistant MDA-MB-231-PR cell lines (Fig. S2B). Cell viability assays were performed on these MDA-MB-231-PR cells following treatment with varying concentrations and different durations of paclitaxel. The results demonstrated that TRIM25 overexpression significantly reduced the sensitivity of MDA-MB-231-PR cells to PTX. Conversely, TRIM25 knockdown produced the opposite effect (Fig. S2C, D). Furthermore, under PTX treatment, TRIM25 overexpression markedly enhanced the colony formation capacity of MDA-MB-231-PR cells and inhibited apoptosis. In contrast, TRIM25 knockdown suppressed colony formation and promoted apoptosis in these cells (Fig. S2E, F). Further analysis revealed that in MDA-MB-231-PR cells, TRIM25 overexpression downregulated the expression level of cleaved-PARP protein upon paclitaxel treatment, while TRIM25 knockdown resulted in a significant upregulation of cleaved-PARP expression in MDA-MB-231-PR cells (Fig. S2G). Collectively, these results demonstrate that TRIM25 plays a critical role in conferring resistance to paclitaxel in breast cancer cells.

### TRIM25 interacts with BRD7

**Fig. 3:**
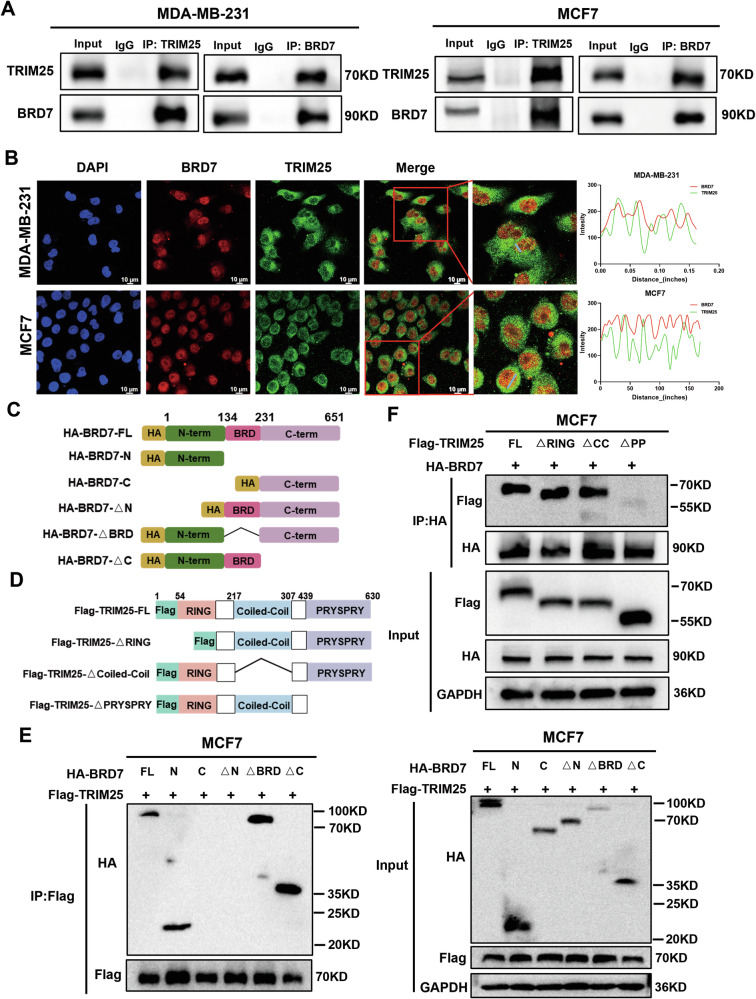
TRIM25 interacts with BRD7. **A** The endogenous interaction between TRIM25 and BRD7 in MDA-MB-231 and MCF7 cells was determined by co-IP assay. **B** Immunofluorescence assays was performed of TRIM25 (green) and BRD7 (red) colocalization in MDA-MB-231 and MCF7 cells. Scale bar, 10 μm. ImageJ was used to quantitatively analyze the co-localization of TRIM25 and BRD7 in breast cancer cells. **C** Schematic of the BRD7 protein full length and deletion mutants. **D** Schematic of the TRIM25 protein, along with the TRIM25 deletion mutants. **E** Co-IP was performed using an anti-Flag antibody to investigate the interaction between BRD7 and TRIM25 in MCF7 cells co-transfected with HA-BRD7 plasmid or HA-BRD7 truncation mutant plasmids together with Flag-TRIM25 plasmids. **F** Co-IP analysis of the interaction between TRIM25 and BRD7 in MCF7 cells co-transfected with Flag-TRIM25 deletion mutants together with HA-BRD7 plasmids.

Since our previous study demonstrated that TRIM25, a latent E3 ubiquitin ligase, is a potential interacting protein of BRD7 [12], we further verified the protein interaction between TRIM25 and BRD7. First, the co-immunoprecipitation (co-IP) assay confirmed that endogenous TRIM25 and BRD7 proteins could be co-immunoprecipitated with each other (Fig. 3A). Next, we determined the colocalization of TRIM25 and BRD7 in BC cells. Immunofluorescence assay in MDA-MB-231 and MCF7 cells showed that TRIM25 was mainly co-located with BRD7 in the nucleus (Fig. 3B). To determine the domain of TRIM25 that interacts with BRD7, we constructed a series of HA-tagged BRD7 deletion mutants and Flag-tagged TRIM25 deletion mutants (Fig. 3C, D). The results of co-IP confirmed that the TRIM25-PRYSPAY domain interacts with the N-terminal region of BRD7 in MCF7 cells (Fig. 3E, F). Taken together, these results indicate that TRIM25 directly interacts with BRD7 in breast cancer cells.

### TRIM25 decreases the stability of the BRD7 protein by regulating K48-linked ubiquitination

**Fig. 4:**
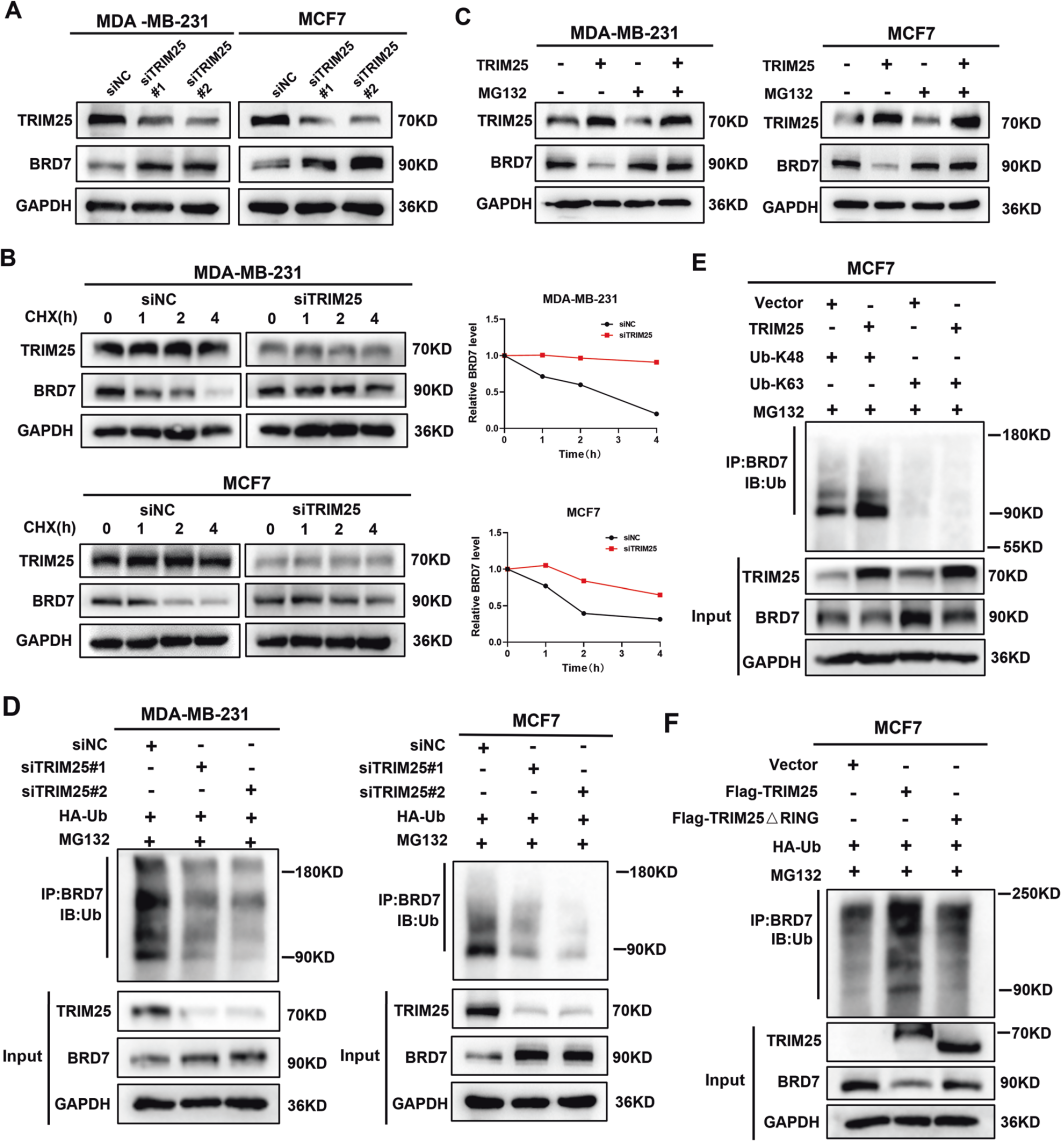
TRIM25 regulates the stability of the BRD7 protein by regulating K48-linked ubiquitination. **A** Western blot analysis of BRD7 protein levels in MDA-MB-231 and MCF7 cells were transfected with siNC, siTRIM25#1, or siTRIM25#2. **B** BC cells transfected with siNC or siTRIM25 incubated with CHX (50 μM) for 0, 1, 2, and 4 h, the BRD7 protein level was detected by western blot. **C** BC cells were transfected with the TRIM25 plasmid or the vector plasmid and further incubated with MG132 (20 μM) for 4 h, the BRD7 protein level was detected by western blot. **D** MDA-MB-231 and MCF7 cells were co-transfected with siNC, siTRIM25#1, or siTRIM25#2 together with HA-Ub, after MG132 (20 μM, 4 h) treatment, the ubiquitination level of BRD7 was detected via IP and western blot analysis. **E** Co-IP and ubiquitination experiment on the changes in BRD7 ubiquitination levels after cotransfection with TRIM25 plasmid and K48-Ub or K63-Ub plasmid in MCF7 cells. **F** Co-IP and ubiquitination experiment was performed to detect the ubiquitination levels of BRD7 in MCF7 cells co-transfected with Flag-TRIM25 plasmid or Flag-TRIM25ΔRING plasmid and HA-UB plasmid.

The interaction between the E3 ubiquitin ligase TRIM25 and BRD7 leads us to presume that TRIM25 decreases BRD7 protein level by ubiquitin-proteasome degradation. Here, we found that the overexpression and knockdown of TRIM25 did not influence BRD7 mRNA levels in either MDA-MB-231 or MCF7 cells (Fig. S3A, B), but TRIM25 negatively regulated the protein expression of BRD7 (Figs. 4A and S3C), indicating that TRIM25 might regulate the level of BRD7 protein by affecting its stability. Furthermore, we utilized cycloheximide (CHX), a protein synthesis inhibitor, to evaluate the influence of TRIM25 on BRD7 protein stability. We found that TRIM25 knockdown significantly increased the half-life of BRD7 (Fig. 4B). Additionally, treatment of BC cells with the proteasome inhibitor MG132 eliminated the effects of TRIM25 overexpression on BRD7 protein, which indicated that TRIM25 downregulation of BRD7 protein in BC cells was mediated by the proteasome pathway (Fig. 4C).

Next, we examined the influence of TRIM25 on BRD7 ubiquitination. Our data showed that the downregulation of TRIM25 markedly reduced the ubiquitination of endogenous BRD7 in BC cells (Fig. 4D), whereas overexpression of TRIM25 enhanced the BRD7 ubiquitination level (Fig. S3D). Next, we investigated the specific subtypes of ubiquitin chains involved in BRD7 ubiquitination mediated by TRIM25. K48-linked and K63-linked ubiquitination stand out as the most prevalent degradation-related mechanisms among ubiquitin-mediated processes. Our result demonstrated that TRIM25 specifically promoted K48-linked ubiquitination of BRD7 (Fig. 4E). Considering that TRIM25 belongs to the TRIM family, most of whose members are RING-type E3 ligases, we sought to determine whether TRIM25 negatively regulated BRD7 protein levels via its E3 ligase activity. We co-transfected MCF7 cells with Flag-TRIM25 plasmid or Flag-TRIM25ΔRING mutant plasmid, along with HA-Ub plasmid. The results showed that the expression of Flag-TIM25, but not that of Flag-TRIM25ΔRING, increased the ubiquitination of BRD7 in MCF7 cells (Fig. 4F). Consistent with this observation, the protein stability of BRD7 was significantly reduced in cells overexpressing TRIM25, but not in those transfected with Flag-TRIM25ΔRING plasmid (Fig. S4A). Thus, these results indicated that TRIM25 promotes ubiquitination of BRD7 in a manner dependent on its RING domain. To identify potential ubiquitination sites on BRD7, we used the UBPRED website to predict six possible ubiquitination sites within the N-terminal domain of BRD7 (K21, K28, K52, K103, K119, and K127) (Fig. S4B). We then mutated lysine (K) residues to arginine (R) residues and evaluated the effects of these mutations on TRIM25-mediated ubiquitination of BRD7. Ubiquitination analysis demonstrated that transient transfection with wild-type HA-BRD7 or K21R, K28R, K52R, K103R, and K127R mutants, but not the K119R mutant, increased TRIM25-mediated BRD7 ubiquitination (Fig. S4C). Collectively, our experiments revealed that TRIM25 facilitated the degradation of BRD7 by inducing K48-linked ubiquitination at the K119 site.

### TRIM25 promotes malignant progression and PTX resistance by degrading BRD7 and activating YB1/Bcl-2 transcription axis

**Fig. 5:**
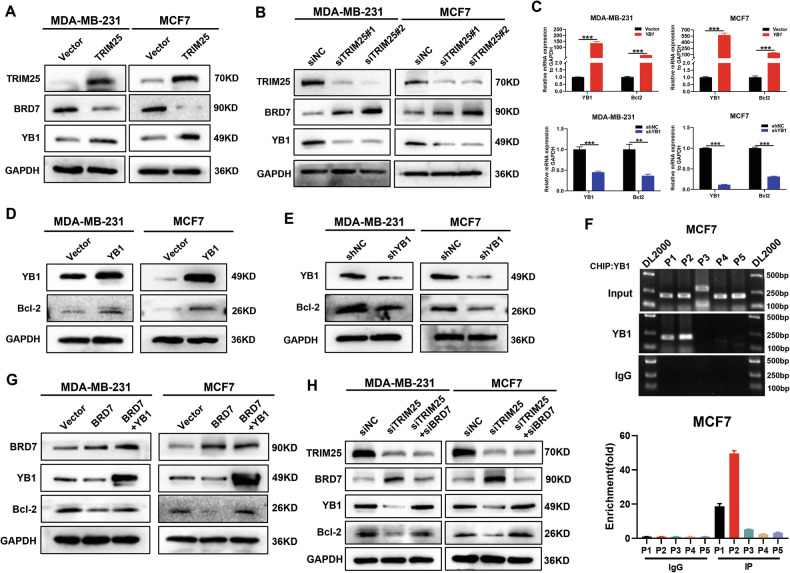
TRIM25 activates YB1/Bcl-2 transcription axis through ubiquitin proteasome pathway-mediated degradation of BRD7 protein in breast cancer cells. **A** MDA-MB-231 and MCF7 cells were transfected with vector or TRIM25 plasmids, the expression level of BRD7 and YB1 were detected by western blot. **B** BC cells were transfected with siNC, siTRIM25#1 or siTRIM25#2, the expression of BRD7 and YB1 were detected by western blot. **C** RT-qPCR analysis was performed to analyze the mRNA expression level of Bcl-2 in YB1 overexpression and knockdown BC cells. **D** Bcl-2 expression was detected by western blot assay under YB1 overexpression. **E** Bcl-2 expression was detected by western blot assay after YB1 knockdown. **F** ChIP-qPCR was used to analyze the enrichment region of YB1 on Bcl-2 promoter fragments. **G** Bcl-2 expression was detected by western blot after BRD7 overexpression or restoring YB1 expression. **H** Western blot was used to detect the alterations of expression of YB1 and Bcl-2 after TRIM25 knockdown or BRD7 restoration. Data are shown as the mean ± SD of at least three independent experiments, and the significant level was identified by **P < 0.01 and ***P < 0.001.

We demonstrated that TRIM25 directly bound to BRD7 and mediated K48-linked ubiquitination and degradation of the BRD7 protein, so we were interested in defining the downstream molecular pathway of the TRIM25/BRD7 axis. In our previous study, Y-box binding protein-1 (YB1), a transcriptional activator, was identified as a novel interacting protein of BRD7 in breast cancer, and BRD7 suppressed cell proliferation and EMT-mediated invasion and metastasis in breast cancer by negatively regulating YB1 [8]. Therefore, we speculated that TRIM25 participated in the malignant progression of breast cancer by regulating the BRD7/YB1 axis. To validate this hypothesis, we detected the protein expression levels of BRD7 and YB1 in TRIM25-overexpressing and TRIM25-knockdown BC cells. Our results showed that overexpression of TRIM25 efficiently reduced the protein levels of BRD7 but promoted the protein levels of YB1. In contrast, TRIM25 knockdown promoted the protein levels of BRD7 but reduced the protein levels of YB1 (Fig. 5A, B), supporting the finding that TRIM25 modulates the BRD7/YB1 axis to promote BC progression.

Bcl-2 has been reported to play an important role in paclitaxel-mediated apoptosis and anti-tumor pathway [25, 26], therefore, it is an important effector protein in paclitaxel-mediated tumor chemotherapy. We used the JASPAR database to predict that there was direct binding of the YB1 protein to the promoter region of Bcl-2, which carries 3 putative YB1-binding sites along the Bcl-2 promoter region, with a relative score higher than 0.85 (Fig. S5A). Since YB1 is a transcription factor, we further explored the effect of YB1 on the protein and mRNA expression of Bcl-2 using qPCR and western blot in MDA-MB-231and MCF7 cells. The results showed that the overexpression of YB1 increased the mRNA and protein levels of Bcl-2, whereas the knockdown of YB1 reduced the protein and mRNA levels of Bcl-2 (Fig. 5C–E). To investigate whether YB1 directly targets the promoter of Bcl-2 in BC cells, the CHIP-qPCR assay was performed. Specifically, to detect whether YB1 could directly bind to the promoter sequence of Bcl-2, we designed three pairs of primers according to the relative score of the potential binding sequence of YB1 and Bcl-2 promoters according to JASPAR database (P1, P2 and P4), meanwhile we designed two pairs of primers as negative control (P3 and P5) according to the sequence of Bcl-2 promoter (Fig. S5A, B). ChIP-qPCR results showed significant enrichment of YB1 at the Bcl-2 promoter regions -782 to -790 and -927 to -919 in MCF7 cells (Fig. 5F). These results proved that YB1 acts as a transcription factor to promote the expression of Bcl-2. In addition, we further tested the effect of BRD7 expression on YB1/Bcl-2 signaling axis by western blot and found that restoring YB1 reversed BRD7-mediated inhibition of Bcl-2 protein (Fig. 5G). Meanwhile, restoring BRD7 expression partially rescued the inhibitory effect of TRIM25 knockdown on YB1 and Bcl-2 (Fig. 5H). Altogether, these data demonstrated that TRIM25, functioning as an E3 ubiquitin ligase, decreased the stability of BRD7 protein through the ubiquitin-proteasome pathway, and inhibited the negative regulation of BRD7 on YB1/Bcl-2 transcriptional axis activity, thus promoting malignant progression and paclitaxel resistance of breast cancer.

### TRIM25 knockdown suppresses cell proliferation and promotes paclitaxel chemosensitivity at least partially through BRD7

**Fig. 6:**
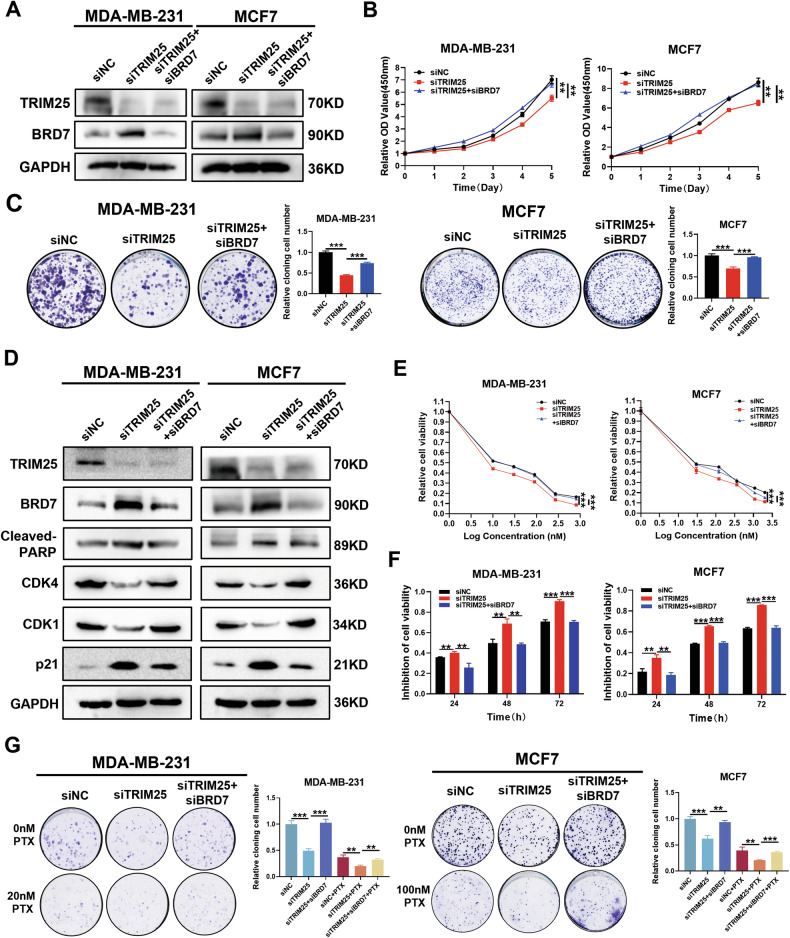
TRIM25 knockdown suppresses cell proliferation and paclitaxel resistance partially through degradation of BRD7. **A** Western blot was performed to detect the expression of TRIM25 and BRD7 in each group. The proliferation (**B**), colony formation (**C**) of the TRIM25 knockdown or BRD7 expression restored BC cells were further detected and analyzed. **D** Western blot was performed to detect the alterations of expression of cell cycle- and apoptosis-related molecules before and after BRD7 restoration. CCK-8 assay was used to evaluate the effects of TRIM25 knockdown and restoring BRD7 expression on the cell viability (**E**) and inhibitory rate of cell growth (**F**) of BC cells treated with PTX. **G** The colony formation capability of TRIM25 knockdown and restoring BRD7 expression BC cells treated with PTX was evaluated by a colony formation assay. Data are shown as the mean ± SD of at least three independent experiments, and the significant level was identified by **P < 0.01 and ***P < 0.001.

To inquire whether the effect of TRIM25 on BC progression and PTX resistance was mediated by BRD7, we carried out several rescue experiments. Firstly, we used siBRD7 to restore the expression of BRD7 in TRIM25-knockdown BC cells, as shown in Fig. 6A, the protein level of BRD7 was successfully restored after transfection with siBRD7 in MDA-MB-231 and MCF7 cells. CCK-8 and colony formation assays showed that TRIM25 downregulation reduced the ability of BC cells to proliferate, while restoration of BRD7 recovered this ability (Fig. 6B, C). Additionally, cell cycle analysis demonstrated that BRD7 restoration reversed the cell cycle arrest in MDA-MB-231 and MCF7 caused by TRIM25 knockdown (Fig. S6A). Our results showed that TRIM25 knockdown decreased the expression of CDK4 and CDK1, and increased the protein levels of P21 as well as cleaved-PARP in both MDA-MB-231 and MCF7 cell lines, whereas the expression levels of these cell cycle and apoptosis-related markers were significantly reversed after BRD7 restoration (Fig. 6D).

In order to further confirm the important role of the TRIM25/BRD7 axis in paclitaxel resistance of breast cancer, we detected the differential expression of TRIM25 and BRD7 between PTX-resistant BC cells and their parent cells. As shown in Fig. S7A, TRIM25 was upregulated and BRD7 was downregulated in MDA-MB-231-PR cells as compared with the parent MDA-MB-231 cells, while TRIM25 knockdown in MDA-MB-231-PR reversed these effects. Cell cycle analysis showed that the ratio of cells in G2/M phase in MDA-MB-231-PR cells was significantly increased compared to that in their parent cells, while TRIM25 knockdown significantly reduced the G2/M phase ratio in MDA-MB-231-PR cells (Fig. S7B). These results suggested that TRIM25 knockdown could reverse the cell cycle progression in PTX-resistant breast cancer cells. In a word, the TRIM25/BRD7 signaling axis is indeed related to the paclitaxel resistance in breast cancer.

Based on these findings, we conducted several rescue experiments to further investigate whether TRIM25 mediates PTX resistance via BRD7. The CCK-8, colony formation, and apoptosis assays were conducted to validate the effects of BRD7 restoration in the TRIM25 knockdown-mediated PTX chemosensitivity in MDA-MB-231 and MCF7 cells. We found that TRIM25 knockdown could increase the PTX chemosensitivity compared with that in the control group, and restoration of BRD7 reversed the PTX chemosensitivity induced by TRIM25 silent expression (Figs. 6E–G, S6B). Similarly, in MDA-MB-231-PR cells, the results of CCK-8 assays, colony formation assays, and apoptosis experiments all demonstrated that restoring the expression of BRD7 reversed the TRIM25 silencing-mediated enhancement of PTX chemosensitization (Fig. S8A–D). Collectively, our data revealed that BRD7 is essential for TRIM25-induced malignant progression and PTX chemoresistance of breast cancer cells.

### TRIM25 promotes tumor growth and PTX resistance through degradation of BRD7 protein in vivo

**Fig. 7:**
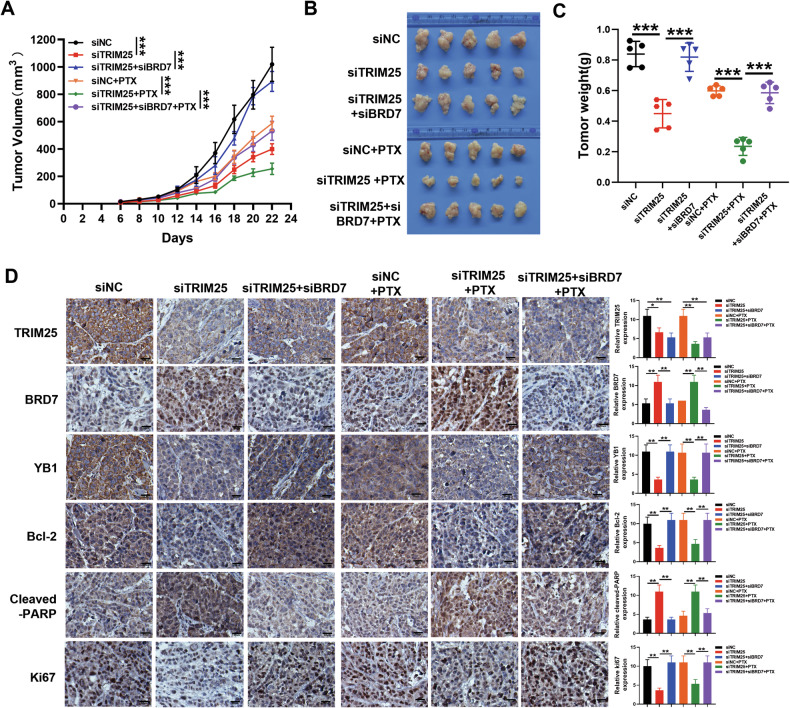
TRIM25 promotes tumor growth and PTX resistance through degradation of BRD7 protein in vivo. **A** Tumor growth curves of the nude mice (*n* = 5 mice for each group). Tumor volume was estimated every 2 days. **B** Representative images of xenograft tumors and tumor weight were calculated for each mouse (**C**). Data are presented as the mean ± SD (*n* = 5 mice for each group). **D** TRIM25, BRD7, YB1, Bcl-2, Ki67, and cleaved-PARP expression in the tumor of nude mice were detected by IHC. Scale bar, 50 μm. Data are shown as the mean ± SD, and the significant level was identified by **P < 0.01 and ***P < 0.001.

We further investigated the effect of the TRIM25/BRD7 axis on tumor growth and PTX resistance in vivo. A nude mouse tumor model was established by using the MCF7 breast cancer cell line, and the mice were divided into six groups, including the siNC, siTRIM25, siTRIM25 plus siBRD7, siNC plus PTX, siTRIM25 plus PTX, and siTRIM25 plus siBRD7 plus PTX groups. For the PTX treatment groups, ten days after subcutaneous implantation, the mice were intraperitoneally injected with PTX (15 mg/kg), and then treated once every two days for a total of four times. The results showed that compared with the siNC group, TRIM25 knockdown slowed tumor growth and reduced tumor weight, whereas the recovery expression of BRD7 reversed the inhibitory effect of TRIM25 knockdown (Fig. 7A–C, S9A). Additionally, in the PTX treatment groups, compared with the untreated groups, the tumor was significantly smaller, indicating the anti-tumor effect of PTX on breast cancer. Meanwhile, compared with the siNC plus PTX group, TRIM25 knockdown significantly increased the anti-tumor effect of PTX, and restoring BRD7 expression could significantly reverse the effect of TRIM25 knockdown on paclitaxel chemotherapy (Fig. 7A–C, S9B). Furthermore, immunohistochemical staining (IHC) indicated that in both the PTX untreated and treated groups, TRIM25 knockdown downregulated the expression of YB1, Ki67, and Bcl-2 but upregulated the expression of BRD7 and cleaved-PARP. Meanwhile, in the PTX untreated groups, TRIM25 knockdown downregulated the expression of CDK4 and CDK1 but upregulated the expression of p21. However, after the expression of BRD7 resumed, the expression of BRD7, YB1, Ki67, CDK4, CDK1, Bcl-2, p21, and cleaved-PARP were partially reversed, which was consistent with the change trend observed in vitro (Fig. 7D, S9C). Taken together, these data validate that TRIM25 promotes breast cancer tumorigenesis and PTX chemotherapy resistance by negatively regulating the stability of BRD7 protein in vivo.

### The predictive value of TRIM25 and BRD7 for the prognosis and diagnosis of clinical breast cancer tissues

**Fig. 8:**
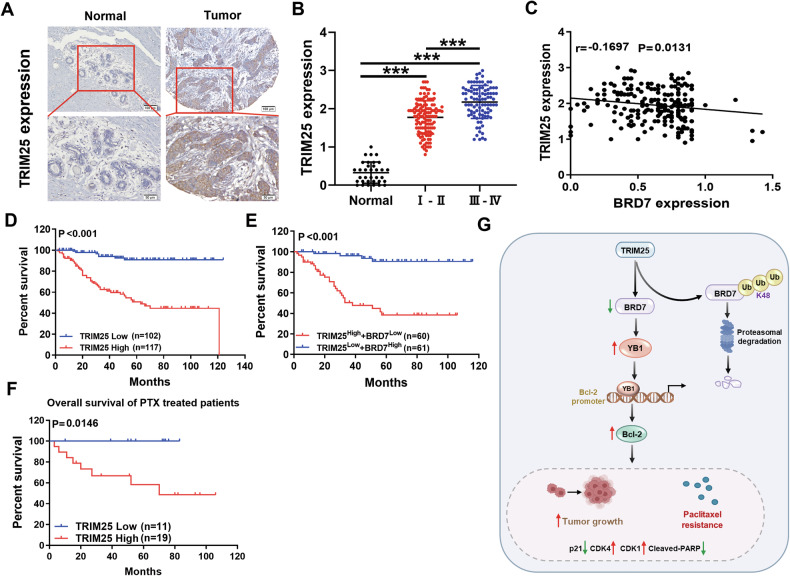
The predictive value of TRIM25 and BRD7 for the prognosis and diagnosis of clinical breast cancer tissues. **A** Representative image of TRIM25 expression in normal breast tissues and BC specimens detected by IHC. Scale bar, 50 μm, 100 μm. **B** IHC analysis to detect the expression of TRIM25 in normal breast tissues and biopsies of BC with different clinical stages. **C** Correlation analysis between TRIM25 and BRD7 protein expression in breast cancer tissue specimens. **D** The Kaplan–Meier curves were used to assess the overall survival of BC patients with high TRIM25 expression and low TRIM25 expression. **E** Kaplan-Meier curves were used to analyze the relationship between the combination expression of TRIM25 and BRD7 and the overall survival of BC patients. **F** The Kaplan–Meier curves were used to detect the relationship between the expression level of TRIM25 and the overall survival of BC patients treated with PTX. **G** Schematic diagram of the molecular mechanism by which TRIM25 promotes breast cancer progression and paclitaxel resistance via ubiquitination-mediated degradation of BRD7 through the YB1-Bcl-2 axis. Data are shown as the mean ± SD, and the significant level was identified by *P < 0.05, **P < 0.01, and ***P < 0.001.

To better understand the potential clinical correlation between TRIM25 and BRD7, we detected the expression of TRIM25 and BRD7 in 34 normal breast tissues and 219 breast cancer biopsies. The results of IHC showed that the expression level of TRIM25 in breast cancer tissues was significantly higher than that in normal breast tissues (Fig. 8A, B). By analyzing the expression of TRIM25 across different clinical stages, we found that its expression in stage III and IV breast cancer tissues was higher than in stage I and II tissues, indicating a positive correlation with disease stage (Fig. 8B). We further explored the expression correlation between BRD7 and TRIM25. As shown in Fig. 8C, the expression level of TRIM25 was negatively correlated with that of BRD7 in BC samples (Pearson correlation coefficient *r* = −0.1697, *P* = 0.0131). Additionally, we evaluated the prognostic value of TRIM25 and BRD7 expression via survival analysis and found that a higher TRIM25 expression was correlated with shorter overall survival (Fig. 8D). Moreover, BC patients with low expression of TRIM25 combined with high expression of BRD7 showed significantly prolonged overall survival (Fig. 8E). Next, we analyzed the correlation between TRIM25 expression and prognosis of breast cancer patients treated with PTX, and found that the breast cancer patients treated with PTX with lower expression of TRIM25 had a longer overall survival (Fig. 8F). In addition, we analyzed the correlation between TRIM25 and BRD7 expression and clinicopathological features in BC patients. The results showed that the expression of TRIM25 was correlated with the clinical stage, tumor size, and distant metastasis of breast cancer patients, but not with the age and lymph node metastasis, while the combined expression of TRIM25 and BRD7 correlated with the clinical stage, tumor size, lymph node metastasis, and distant metastasis of breast cancer patients (Table 3). Overall, these data identified that the TRIM25/BRD7 axis could guide the early diagnosis and treatment in breast cancer patients and provide a potential therapeutic target for breast cancer therapy.

**Table 3:** The association between TRIM25, BRD7 expression, and clinicopathologic features of breast cancer.

Variables	TRIM25 expression				TRIM25/BRD7 expression		
Features	TRIM25^L^	TRIM25^H^	*P*		TRIM25^L^BRD7^H^	TRIM25^H^BRD7^L^	*P*
	(102)	(117)			(61)	(60)	
Age (Y [%])							
≤46	51 (50.00%)	60 (49.43%)	0.8499		27 (44.26%)	27 (45.00%)	0.9350
>46	51 (50.00%)	57 (50.57%)			34 (55.74%)	33 (55.00%)	
Tumor size (*n*[%])							
T1-2	72 (70.59%)	52 (44.44%)	0.0001 ***		43 (70.49%)	24 (40.00%)	0.0007 ***
T3-4	30 (29.41%)	64 (54.70%)			18 (29.51%)	36 (60.00%)	
*N* (*n*[%])							
Absent	30 (29.41%)	23 (19.66%)	0.9998		19 (31.15%)	9 (15.00%)	0.0352 **
Present	70 (70.59%)	93 (79.49%)			42 (68.85%)	51 (85.00%)	
M (*n*[%])							
Absent	101 (99.02%)	97 (82.91%)	0.0001 ***		60 (98.36%)	49 (81.67%)	0.0021 **
Present	1 (1.08%)	18 (15.39%)			1 (1.64%)	11 (18.33%)	
Stage (*n*[%])							
Ⅰ-Ⅱ	76 (74.51%)	50 (42.73%)	*P* < 0.001 ***		45 (73.77%)	23 (38.33%)	0.0109*
Ⅲ-Ⅳ	26 (25.49%)	66 (56.41%)			16 (26.23%)	37 (61.67%)	

In 219 patients, Tumor size, lymph node metastasis, and clinical stage were not detected in one of the patients with high TRIM25 expression; distant metastasis was not detected in two of the patients with high TRIM25 expression. The median of TRIM25 expression or BRD7 expression was used as the cutoff value. *Y* Year, *TNM* Tumor-node-metastases, *H* High expression, *L* Low expression, *P P* values of two-sided χ2 test, % The ratio of the number of samples to the total number of samples per column, * *P* < 0.05, ** *P* < 0.01, *** *P* < 0.001.

## Discussion

Previous studies have shown the key role of BRD7 in suppressing cell proliferation, blocking cell cycle processes, inducing cell apoptosis, suppressing cell migration and invasion, and promoting sensitivity to PTX chemotherapy in breast cancer [7–9]. The dysregulation of BRD7 expression in breast cancer is an important mechanism leading to tumor occurrence, development, and PTX resistance. Our previous study showed that BRD7 was an unstable protein in breast cancer, and the ubiquitin-proteasome pathway was involved in the regulation of BRD7 protein instability [12]. To further reveal the mechanism of BRD7 in tumor development and PTX chemotherapy sensitization in breast cancer, we screened and identified a potential E3 ubiquitin ligase TRIM2,5 interacting with BRD7 by IP-MS. Various studies have pointed out that TRIM25, as an E3 ubiquitin ligase, promotes ubiquitination degradation of substrates through ubiquitin-proteasome-dependent pathway, thus playing a crucial role in the occurrence and development of tumors. For example, in non-small cell lung cancer (NSCLC), TRIM25 mediates the K63-linked ubiquitination of PTEN at the K266 site, and promotes the proliferation of NSCLC cells and tumor growth by activating AKT/mTOR signaling pathway [33]. TRIM25 inhibits the invasion and migration of hepatocellular carcinoma cells by targeting the ubiquitination and degradation of Metastasis-Associated 1 (MTA1) protein [34]. In our study, we demonstrated that TRIM25 directly interacted with BRD7 protein, and the PRYSPRY domain of TRIM25 and the N-terminus of BRD7 protein were the molecular basis for the direct binding between TRIM25 and BRD7. Additionally, TRIM25 promoted K48-linked ubiquitination of BRD7 at the K119 site. Thus, we have shown that TRIM25 negatively regulates the stability of BRD7 protein through the ubiquitin-proteasome pathway, and confirmed the direct binding domains of TRIM25 and BRD7, which provide the molecular basis for further development of targeted therapy for the treatment of breast cancer.

Evidence shows that TRIM25 is low-expressed in many tumors, including liver cancer, lung cancer, and ovarian cancer, and is closely related to the poor prognosis of tumor patients [33, 35, 36]. Moreover, TRIM25 has been proven to be an important determinant of the breast cancer malignant process and an effective prognostic factor for breast cancer patients [37–40]. For example, TRIM25 mediates the degradation of ATBF1 protein induced by estrogen, promoting the proliferation of breast cancer cells [41], and TRIM25 also degrades 14-3-3σ protein, promoting tumor growth in breast cancer [42]. Consistent with these results, we confirmed that TRIM25 significantly promoted cell proliferation and tumor growth in breast cancer. Meanwhile, we confirmed that the expression of TRIM25 in breast cancer patients was significantly upregulated, and its expression level positively correlated with clinical stages and poor prognosis of BC patients, indicating that TRIM25 is a potential oncogene in breast cancer. Previous studies have identified TRIM25’s role in promoting chemotherapy resistance in tumors. TRIM25 is involved in the malignant progression of lung cancer and colorectal cancer by increasing the sensitivity of adriamycin-mediated chemotherapy [16, 17]. Qin et al. reported that TRIM25 promoted cisplatin resistance in lung cancer cells by down-regulating the expression of 14-3-3δ and P53 [43]. In the present study, we revealed the important role of TRIM25 in promoting PTX-mediated chemotherapy resistance in breast cancer, demonstrated both in vitro and in vivo. Therefore, these findings suggest that targeting TRIM25 may be a promising strategy to enhance the PTX chemotherapy response in breast cancer.

Our results confirmed that TRIM25 was involved in the malignant progression of breast cancer and PTX resistance, and that TRIM25 participates in the regulation of ubiquitination level and protein degradation of BRD7 through the ubiquitin-proteasome pathway. Therefore, we suspect that BRD7 mediates the process of TRIM25 promoting the occurrence and development of breast cancer and PTX resistance. The results demonstrated that the restoration of BRD7 effectively rescued the inhibition of proliferation, tumor growth, and paclitaxel chemosensitivity mediated by TRIM25 silencing in vitro and in vivo, emphasizing that TRIM25 regulated the malignant progression and PTX resistance of BC in a BRD7-dependent manner. In addition, our previous study proved that BRD7 reduced the stability of YB1 protein through the ubiquitin-proteasome pathway and inhibited the malignant progression of breast cancer [8]. Furthermore, we found that a binding site for YB1 exists in the promoter region of Bcl-2 using the JASPAR database; Bcl-2 is an apoptosis inhibitor and an important effector protein in the process of paclitaxel chemotherapy. Therefore, we suspected that TRIM25, as an upstream regulator of BRD7, could regulate the YB1/Bcl-2 signal axis mediated by BRD7 and participate in the malignant progression and PTX resistance of BC. Subsequently, our results showed that YB1 acted as a transcription factor to promote the expression of Bcl-2, and BRD7 restoration partially rescued the downregulation of YB1 protein level and Bcl-2 protein level mediated by TRIM25 knockdown. These data suggested that TRIM25 decreased the stability of BRD7 protein through the ubiquitin-proteasome pathway, thereby activating the YB1/Bcl-2 transcription axis and resulting in the malignant progression and PTX resistance of breast cancer. In addition to mediating PTX resistance in breast cancer cells via the regulation of YB1-Bcl-2 signal axis, TRIM25 is reported to promote the nuclear translocation of transcription factor EB (TFEB) and transcription of autophagy-related genes by increasing ubiquitination of TFEB, thereby decreasing chemosensitivity to PTX in TNBC [18]. Furthermore, a study showed that TRIM25 could affect the subcellular translocation of p53, which was relevant for resistance towards docetaxel [44]. The above findings prove that TRIM25 plays a vital role in the chemotherapy resistance of paclitaxel in breast cancer through various mechanisms. In short, our research provides a new insight into the mechanism of TRIM25 participating in paclitaxel chemotherapy resistance. Accordingly, targeting the TRIM25/BRD7/YB1/Bcl-2 signal axis may be a potential molecular target for early diagnosis of breast cancer and promoting the sensitization of PTX in clinical breast cancer patients.

Herein, we further clarify the clinical value and significance of the expression of TRIM25 and BRD7 in patients with breast cancer. Several articles have reported that TRIM25 can be used as a molecular target for clinical diagnosis and prognosis of tumors. TRIM25 has been proven to be related to the differentiation degree, TNM staging, and lymph node metastasis of non-small cell lung cancer, and is related to the poor prognosis of patients [33, 45]. The combination of RIG-I and TRIM25 can predict the prognosis of breast cancer patients with estrogen receptor-positive [46], and the high expression of TRIM25 is related to the poor prognosis of patients with gastric cancer [47]. Our results confirmed that TRIM25 was highly expressed in breast cancer tissues, and its high expression was positively correlated with advanced clinical stages and poor survival in BC patients. Furthermore, BC patients with higher TRIM25 levels showed a poorer response to PTX treatment and a poor prognosis. Additionally, TRIM25 expression was negatively correlated with BRD7 expression, and the combination of BRD7 low expression and TRIM25 high expression correlated with a poor prognosis of BC patients. These findings indicate that the TRIM25/BRD7 signaling axis can be used as a potential target to guide the early diagnosis and treatment of breast cancer patients, and provide a promising strategy for overcoming resistance to PTX and improving the prognosis of breast cancer patients.

## Conclusion

Taken together, the present study shows that TRIM25 works as an oncogene and promotes PTX resistance in breast cancer. Further studies showed that TRIM25 directly interacts with BRD7 and then increases the K48-linked ubiquitination of BRD7 at the K119 site, and subsequently results in activating the YB1/Bcl-2 signal axis (Fig. 8G). The recovery of BRD7 expression reversed the inhibitory effects of TRIM25 knockdown on the proliferation, tumor growth, and paclitaxel resistance of BC cells in vitro and in vivo. In addition, TRIM25 is highly expressed in BC tissues and correlates with poor survival prognosis and clinicopathological features. Consequently, these findings reveal that TRIM25 promotes ubiquitination-mediated degradation of BRD7 and promotes breast cancer progression and paclitaxel resistance via the YB1-Bcl-2 axis, which provides a new prognostic predictor and therapeutic target for breast cancer.

## Supplementary information

Supplementary data

checklist

Revised Original blots and gels in this manuscript

## Data Availability

All data generated or analyzed during this study are included in this published article and its supplementary information files.

## References

[1] Siegel RL, Miller KD, Wagle NS, Jemal A. Cancer Statistics, 2023. CA Cancer J Clin. 2023;73:17–48. 36633525 10.3322/caac.21763

[2] Chen X, Wu W, Jeong JH, Rokavec M, Wei R, Feng S, et al. Cytokines-activated nuclear IKKalpha-FAT10 pathway induces breast cancer tamoxifen-resistance. Sci China Life Sci. 2024;67:1413–26. 38565741 10.1007/s11427-023-2460-0

[3] Abu Samaan TM, Samec M, Liskova A, Kubatka P, Busselberg D. Paclitaxel’s mechanistic and clinical effects on breast cancer. Biomolecules. 2019;9:789. 10.3390/biom9120789 PMC6995578 31783552

[4] Crown J, O’Leary M, Ooi WS. Docetaxel and paclitaxel in the treatment of breast cancer: a review of clinical experience. Oncologist. 2004;9:24–32. 15161988 10.1634/theoncologist.9-suppl_2-24

[5] Keklikoglou I, Cianciaruso C, Guc E, Squadrito ML, Spring LM, Tazzyman S, et al. Chemotherapy elicits pro-metastatic extracellular vesicles in breast cancer models. Nat Cell Biol. 2019;21:190–202. 30598531 10.1038/s41556-018-0256-3 PMC6525097

[6] Kaeser MD, Aslanian A, Dong MQ, Yates JR 3rd, Emerson BM. BRD7, a novel PBAF-specific SWI/SNF subunit, is required for target gene activation and repression in embryonic stem cells. J Biol Chem. 2008;283:32254–63. 18809673 10.1074/jbc.M806061200 PMC2583284

[7] Ma J, Niu W, Wang X, Zhou Y, Wang H, Liu F, et al. Bromodomain‑containing protein 7 sensitizes breast cancer cells to paclitaxel by activating Bcl2‑antagonist/killer protein. Oncol Rep. 2019;41:1487–96. 30592293 10.3892/or.2018.6951 PMC6365691

[8] Niu W, Luo Y, Zhou Y, Li M, Wu C, Duan Y, et al. BRD7 suppresses invasion and metastasis in breast cancer by negatively regulating YB1-induced epithelial-mesenchymal transition. J Exp Clin Cancer Res. 2020;39:30. 32028981 10.1186/s13046-019-1493-4 PMC7006413

[9] Luo Y, Wang X, Niu W, Zhou Y, Li M, Ma J, et al. BRD7 stabilizes P53 via Dephosphorylation of MDM2 to inhibit tumor growth in breast cancer harboring Wild-type P53. J Cancer. 2022;13:1436–48. 35371302 10.7150/jca.67447 PMC8965117

[10] Xiao Z, Chang JG, Hendriks IA, Sigurethsson JO, Olsen JV, Vertegaal AC. System-wide analysis of SUMOylation dynamics in response to replication stress reveals novel small ubiquitin-like modified target proteins and acceptor lysines relevant for genome stability. Mol Cell Proteom. 2015;14:1419–34. 10.1074/mcp.O114.044792 PMC4424410 25755297

[11] Olsen JV, Blagoev B, Gnad F, Macek B, Kumar C, Mortensen P, et al. Global, in vivo, and site-specific phosphorylation dynamics in signaling networks. Cell. 2006;127:635–48. 17081983 10.1016/j.cell.2006.09.026

[12] Xue C, Meng H, Niu W, Li M, Wei J, Chen S, et al. TRIM28 promotes tumor growth and metastasis in breast cancer by targeting the BRD7 protein for ubiquitination and degradation. Cell Oncol. 2024;47:1973–93. 10.1007/s13402-024-00981-3 PMC12974028 39222175

[13] Martin-Vicente M, Medrano LM, Resino S, Garcia-Sastre A, Martinez I. TRIM25 in the regulation of the antiviral innate immunity. Front Immunol. 2017;8:1187. 29018447 10.3389/fimmu.2017.01187 PMC5614919

[14] Choudhury NR, Heikel G, Michlewski G. TRIM25 and its emerging RNA-binding roles in antiviral defense. Wiley Interdiscip Rev RNA. 2020;11:e1588. 31990130 10.1002/wrna.1588

[15] Heikel G, Choudhury NR, Michlewski G. The role of Trim25 in development, disease and RNA metabolism. Biochem Soc Trans. 2016;44:1045–50. 27528750 10.1042/BST20160077

[16] Qin Y, Cui H, Zhang H. Overexpression of TRIM25 in lung cancer regulates tumor cell progression. Technol Cancer Res Treat. 2016;15:707–15. 26113559 10.1177/1533034615595903

[17] Nasrullah U, Haeussler K, Biyanee A, Wittig I, Pfeilschifter J, Eberhardt W Identification of TRIM25 as a negative regulator of Caspase-2 Expression reveals a novel target for sensitizing colon carcinoma cells to intrinsic apoptosis. Cells. 2019;8:1622. 10.3390/cells8121622 PMC6952940 31842382

[18] Zheng B, Qian F, Wang X, Wang Y, Zhou B, Fang L. Neddylation activated TRIM25 desensitizes triple-negative breast cancer to paclitaxel via TFEB-mediated autophagy. J Exp Clin Cancer Res. 2024;43:177. 10.1186/s13046-024-03085-w PMC11201311 38926803

[19] Chen Y, Xu X, Ding K, Tang T, Cai F, Zhang H, et al. TRIM25 promotes glioblastoma cell growth and invasion via regulation of the PRMT1/c-MYC pathway by targeting the splicing factor NONO. J Exp Clin Cancer Res. 2024;43:39. 38303029 10.1186/s13046-024-02964-6 PMC10835844

[20] Zhou S, Peng J, Xiao L, Zhou C, Fang Y, Ou Q, et al. TRIM25 regulates oxaliplatin resistance in colorectal cancer by promoting EZH2 stability. Cell Death Dis. 2021;12:463. 33966039 10.1038/s41419-021-03734-4 PMC8106682

[21] Wu J, Lee C, Yokom D, Jiang H, Cheang MC, Yorida E, et al. Disruption of the Y-box binding protein-1 results in suppression of the epidermal growth factor receptor and HER-2. Cancer Res. 2006;66:4872–9. 16651443 10.1158/0008-5472.CAN-05-3561

[22] Jiang, Qiu D, Peng T, Li J, Tala S, Ren W, et al. YB-1 is a positive regulator of KLF5 transcription factor in basal-like breast cancer. Cell Death Differ. 2022;29:1283–95. 35022570 10.1038/s41418-021-00920-x PMC9177637

[23] Mouneimne G, Brugge JS. YB-1 translational control of epithelial-mesenchyme transition. Cancer Cell. 2009;15:357–9. 19411064 10.1016/j.ccr.2009.04.006

[24] Czabotar PE, Garcia-Saez AJ. Mechanisms of BCL-2 family proteins in mitochondrial apoptosis. Nat Rev Mol Cell Bio. 2023;24:732–48. 37438560 10.1038/s41580-023-00629-4

[25] Tabuchi Y, Matsuoka J, Gunduz M, Imada T, Ono R, Ito M, et al. Resistance to paclitaxel therapy is related with Bcl-2 expression through an estrogen receptor mediated pathway in breast cancer. Int J Oncol. 2009;34:313–9. 19148464

[26] Callagy GM, Pharoah PD, Pinder SE, Hsu FD, Nielsen TO, Ragaz J, et al. Bcl-2 is a prognostic marker in breast cancer independently of the Nottingham Prognostic Index. Clin Cancer Res. 2006;12:2468–75. 16638854 10.1158/1078-0432.CCR-05-2719

[27] Haldar S, Chintapalli J, Croce CM. Taxol induces Bcl-2 phosphorylation and death of prostate cancer cells. Cancer Res. 1996;56:1253–5. 8640809

[28] Zhao R, Liu Y, Wu C, Li M, Wei Y, Niu W, et al. BRD7 Promotes cell proliferation and tumor growth through stabilization of c-Myc in colorectal cancer. Front Cell Dev Biol. 2021;9:659392. 10.3389/fcell.2021.659392 PMC8181413 34109174

[29] Stiekema M, Ramaekers FCS, Kapsokalyvas D, van Zandvoort M, Veltrop RJA, Broers JLV. super-resolution imaging of the A- and B-Type Lamin networks: a comparative study of different fluorescence labeling procedures. Int J Mol Sci. 2021;22:10194. 10.3390/ijms221910194 PMC8508656 34638534

[30] O’Brien CE, Bonanno L, Zhang H, Wyss-Coray T. Beclin 1 regulates neuronal transforming growth factor-beta signaling by mediating recycling of the type I receptor ALK5. Mol Neurodegener. 2015;10:69. 26692002 10.1186/s13024-015-0065-0 PMC4687091

[31] Li M, Wei Y, Liu Y, Wei J, Zhou X, Duan Y, et al. BRD7 inhibits enhancer activity and expression of BIRC2 to suppress tumor growth and metastasis in nasopharyngeal carcinoma. Cell Death Dis. 2023;14:121. 36788209 10.1038/s41419-023-05632-3 PMC9929072

[32] Li M, Wei J, Xue C, Chen S, Zhou X, Zheng L, et al. BRD7 enhances the radiosensitivity of nasopharyngeal carcinoma cells by negatively regulating USP5/METTL3 axis-mediated homologous recombination repair. Int J Biol Sci. 2024;20:6130–45. 39664566 10.7150/ijbs.100833 PMC11628346

[33] He YM, Zhou XM, Jiang SY, Zhang ZB, Cao BY, Liu JB, et al. TRIM25 activates AKT/mTOR by inhibiting PTEN via K63-linked polyubiquitination in non-small cell lung cancer. Acta Pharm Sin. 2022;43:681–91. 10.1038/s41401-021-00662-z PMC8888698 33931764

[34] Zang HL, Ren SN, Cao H, Tian XF. The ubiquitin ligase TRIM25 inhibits hepatocellular carcinoma progression by targeting metastasis-associated 1 protein. IUBMB Life. 2017;69:795–801. 28861931 10.1002/iub.1661

[35] Liu Y, Tao S, Liao L, Li Y, Li H, Li Z, et al. TRIM25 promotes the cell survival and growth of hepatocellular carcinoma through targeting Keap1-Nrf2 pathway. Nat Commun. 2020;11:348. 31953436 10.1038/s41467-019-14190-2 PMC6969153

[36] Sakuma M, Akahira J, Suzuki T, Inoue S, Ito K, Moriya T, et al. Expression of estrogen-responsive finger protein (Efp) is associated with advanced disease in human epithelial ovarian cancer. Gynecol Oncol. 2005;99:664–70. 16140366 10.1016/j.ygyno.2005.07.103

[37] Walsh LA, Alvarez MJ, Sabio EY, Reyngold M, Makarov V, Mukherjee S, et al. An integrated systems biology approach identifies TRIM25 as a key determinant of breast cancer metastasis. Cell Rep. 2017;20:1623–40. 28813674 10.1016/j.celrep.2017.07.052 PMC5985663

[38] Thomson SD, Ali S, Pickles L, Taylor J, Pace PE, Lymboura M, et al. Analysis of estrogen-responsive finger protein expression in benign and malignant human breast. Int J Cancer. 2001;91:152–8. 11146438 10.1002/1097-0215(200002)9999:9999<::aid-ijc1032>3.0.co;2-y

[39] Sato W, Ikeda K, Gotoh N, Inoue S, Horie K. Efp promotes growth of triple-negative breast cancer cells. Biochem Biophys Res Commun. 2022;624:81–8. 35940131 10.1016/j.bbrc.2022.07.071

[40] Ueyama K, Ikeda K, Sato W, Nakasato N, Horie-Inoue K, Takeda S, et al. Knockdown of Efp by DNA-modified small interfering RNA inhibits breast cancer cell proliferation and in vivo tumor growth. Cancer Gene Ther. 2010;17:624–32. 20467453 10.1038/cgt.2010.19

[41] Dong XY, Fu X, Fan S, Guo P, Su D, Dong JT. Oestrogen causes ATBF1 protein degradation through the oestrogen-responsive E3 ubiquitin ligase EFP. Biochem J. 2012;444:581–90. 22452784 10.1042/BJ20111890 PMC3754848

[42] Urano T, Saito T, Tsukui T, Fujita M, Hosoi T, Muramatsu M, et al. Efp targets 14-3-3 sigma for proteolysis and promotes breast tumour growth. Nature. 2002;417:871–5. 12075357 10.1038/nature00826

[43] Qin X, Qiu F, Zou Z. TRIM25 is associated with cisplatin resistance in non-small-cell lung carcinoma A549 cell line via downregulation of 14-3-3s. Biochem Biophys Res Commun. 2017;493:568–72. 28867193 10.1016/j.bbrc.2017.08.151

[44] Takayama KI, Suzuki T, Tanaka T, Fujimura T, Takahashi S, Urano T, et al. TRIM25 enhances cell growth and cell survival by modulating p53 signals via interaction with G3BP2 in prostate cancer. Oncogene. 2018;37:2165–80. 29379164 10.1038/s41388-017-0095-x

[45] Han Q, Cheng P, Yang H, Liang H, Lin F. Altered expression of microRNA-365 is related to the occurrence and development of non-small-cell lung cancer by inhibiting TRIM25 expression. J Cell Physiol. 2019;234:22321–30. 31099423 10.1002/jcp.28798

[46] Sato J, Azuma K, Kinowaki K, Ikeda K, Ogura T, Takazawa Y, et al. Combined Use of Immunoreactivities of RIG-I with Efp/TRIM25 for predicting prognosis of patients with estrogen receptor-positive breast cancer. Clin Breast Cancer. 2021;21:399–407.e2. 33386231 10.1016/j.clbc.2020.12.001

[47] Zhu Z, Wang Y, Zhang C, Yu S, Zhu Q, Hou K, et al. TRIM25 blockade by RNA interference inhibited migration and invasion of gastric cancer cells through TGF-β signaling. Sci Rep. 2016;6:19070. 26754079 10.1038/srep19070 PMC4709557

