# Supplementary material for: TRIM25 degrades BRD7 protein stability through the ubiquitin proteasome pathway to promote breast cancer progression and paclitaxel resistance by activating YB1/Bcl-2 transcription axis
Source: Cell Death Dis. 2025 Nov 28;16(1):872. doi: 10.1038/s41419-025-08140-8 (PMC12663559; doi:10.1038/s41419-025-08140-8)
Supplement: Supplementary file 1 — Supplementary data [file 41419_2025_8140_MOESM1_ESM.pdf]

Supplementary data

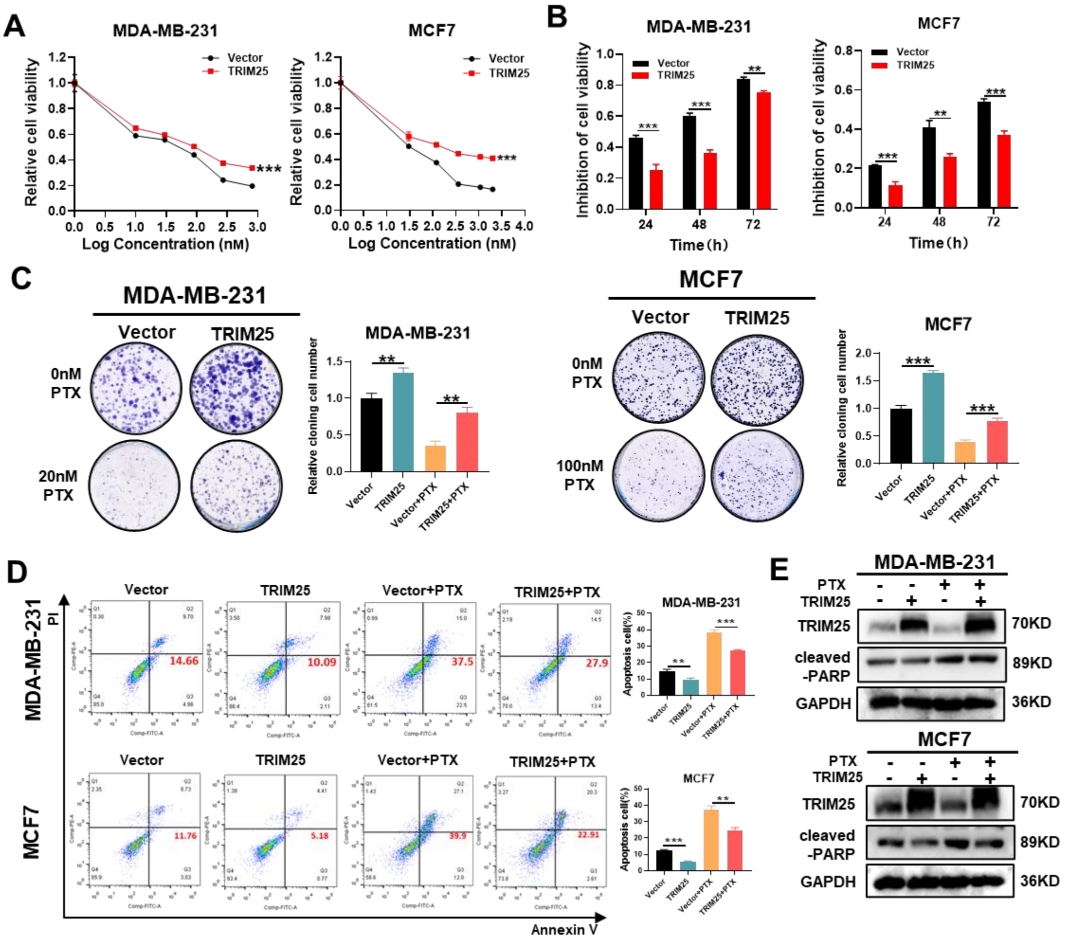

Figure. S1 TRIM25 overexpression increases the chemotherapy resistance of BC cells to PTX. CCK-8 assay was used to detect the cell viability (**A**) and the inhibition rate (**B**) of TRIM25 overexpressed BC cells treated with PTX. **C.** Colony formation ability was assessed through colony formation assay of TRIM25 overexpressed BC cells treated with PTX. **D.** MDA-MB-231 and MCF7 cells were transfected with Vector or TRIM25, and then treated with PTX for 48 h (MDA-MB-231: 20 nM, MCF7: 100 nM), and the apoptosis ratio was detected through flow cytometry. **E.** BC cells were treated with or without PTX (MDA-MB-231: 20 nM, MCF7: 100 nM) for 24 h, cleaved-PARP protein levels in TRIM25 overexpressed BC cells were detected by western blot analysis. Data are shown as the mean ± SD of at least three independent experiments, and the significant level was identified by $^{**}P< 0.01$, and $^{***}P < 0.001$.

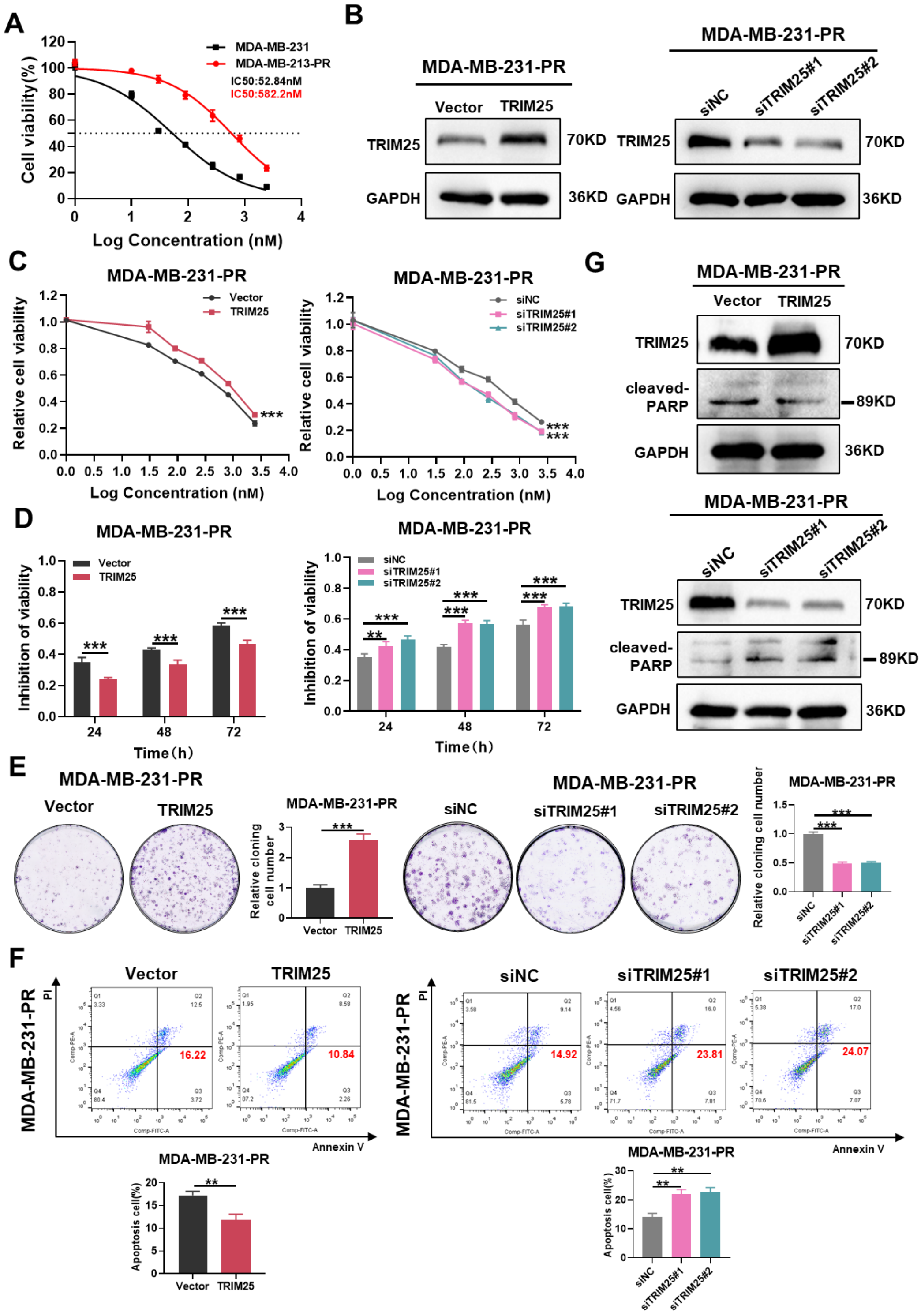

Figure. S2 TRIM25 reduces chemosensitivity of paclitaxel-resistant BC cell lines to paclitaxel. **A.** Cell viability was detected by the CCK-8 method, and the dose–response curves of paclitaxel in parental and resistant cells were plotted. The IC50 value was calculated according to the fitting curves. **B.** Western blot was used to detect the over-expression or down-regulation of TRIM25 in MDA-MB-231-PR cells. **C.** CCK-8 assay was used to detect the cell viability of TRIM25 overexpressed or TRIM25 knockdown

MDA-MB-231-PR cells treated with PTX (0 nM, 1 nM, 30 nM, 90 nM, 270 nM, 810 nM, 2430 nM). **D.** CCK-8 assay was used to detect the cell inhibition rate of TRIM25 overexpressed or TRIM25 knockdown MDA-MB-231-PR cells treated with PTX (200 nM). **E.** Colony formation ability was assessed through colony formation assay of TRIM25 overexpressed or knockdown MDA-MB-231-PR cells treated with PTX (200 nM). **F.** The apoptosis rate induced by PTX in MDA-MB-231-PR cells with over-expression or silent expression of TRIM25 was detected through flow cytometry. **G.** MDA-MB-231-PR cells were treated with PTX (200 nM) for 24 h, cleaved-PARP protein levels in TRIM25 overexpressed or knockdown MDA-MB-231-PR cells were detected by western blot analysis. PR, paclitaxel resistance. Data are shown as the mean ± SD of at least three independent experiments, and the significant level was identified by $^{**}P < 0.01$, and $^{***}P < 0.001$.

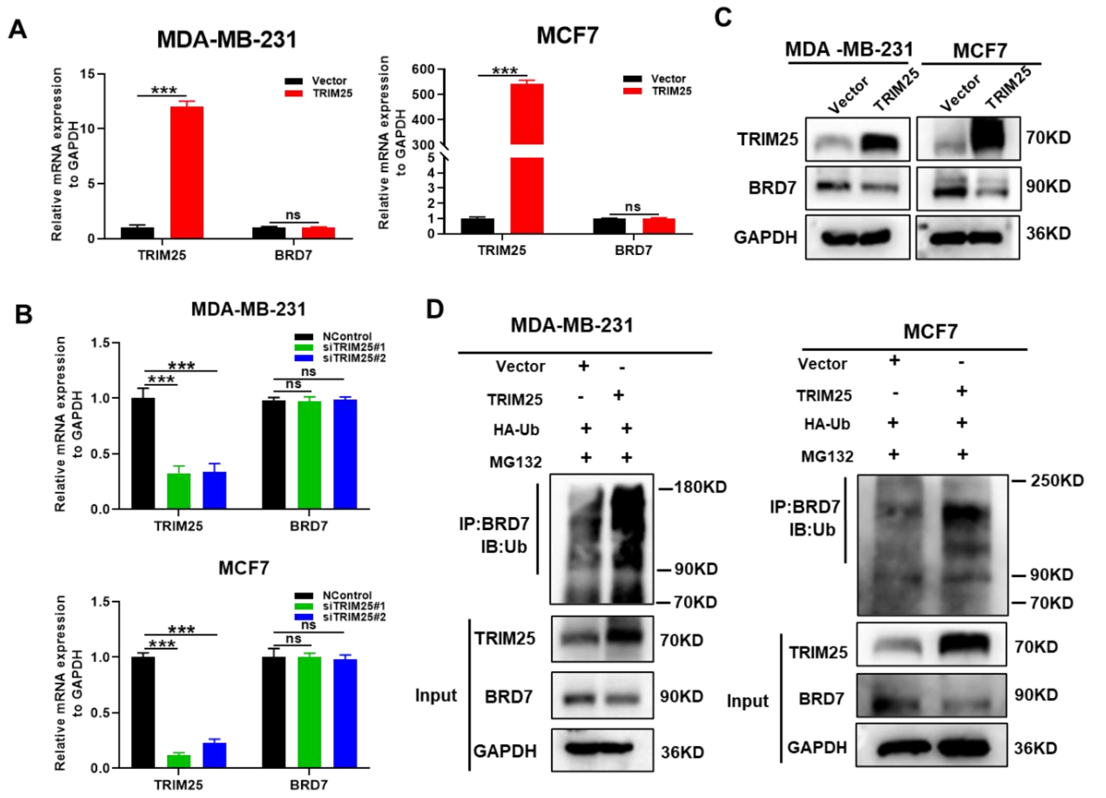

Figure.S3 TRIM25 overexpression inhibits BRD7 protein level and increases BRD7 ubiquitination. **A.** RT-qPCR validation of TRIM25 and BRD7 expression in BC cells transfected with vector or TRIM25 plasmids. **B.** RT-qPCR validation of TRIM25 and BRD7 expression in breast cancer cells transfected with siTRIM25. **C.** Western blot analysis of TRIM25 and BRD7 in MDA-MB-231 and MCF7 cells transfected with vector or TRIM25 plasmids. **D.** The effect of TRIM25 overexpression on the ubiquitination level of BRD7 in MDA-MB-231 and MCF7 cells treated with MG132 (20 µM) for 4 h. Data are shown as the mean ± SD of at least three independent experiments, and the significant level was identified by ns, no significance, $*P < 0.05$, $**P< 0.01$, and $***P < 0.001$

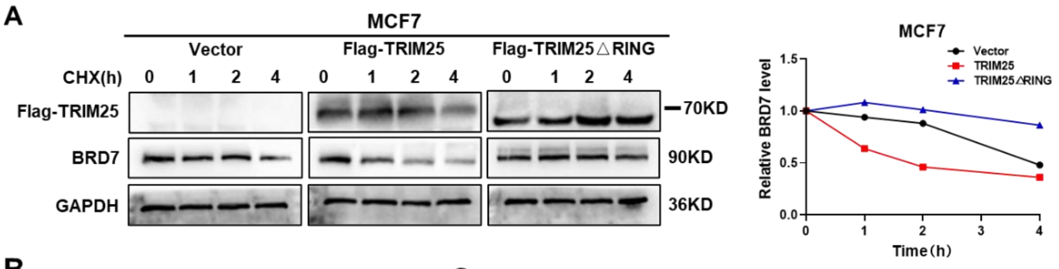

B

Predicted Ubiquitination sites of BRD7		
Peptide	Position	Score
LYEEYVEKPLKLVLK	21	0.64
KPLKLVLKVGGNEVT	28	0.68
DSSLFEDKNDHDKHK	52	0.92
RVENEAEKDLQCHAP	103	0.94
RLDLPPEKPLTSSLA	119	0.85
PLTSSLAKQEEVEQT	127	0.92

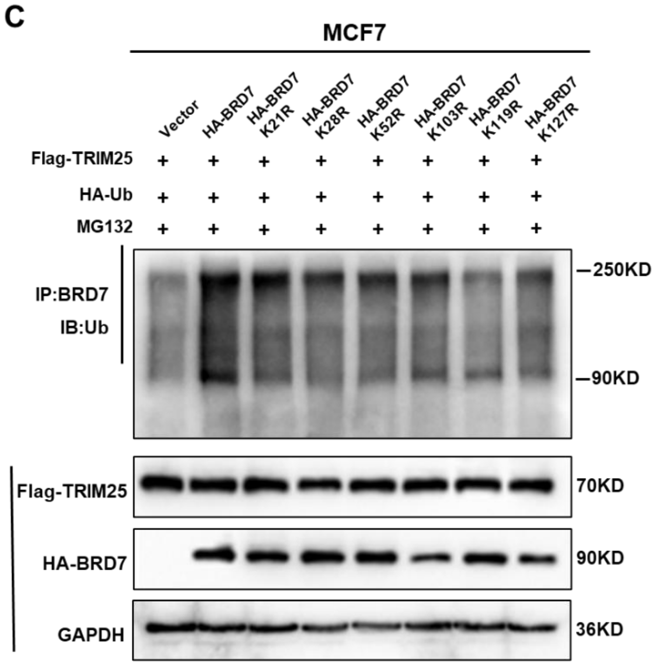

Figure. S4 TRIM25 regulates the stability of the BRD7 protein relies on the RING domain and facilitates the degradation of BRD7 at the K119 site. **A.** Western blot assay was performed to detect the BRD7 protein level transfected with Flag-TRIM25 plasmid or Flag-TRIM25ΔRING plasmid in MCF7 cells incubated with CHX (50 μM) for 0, 1, 2, and 4 h. **B.** The UbPred website (http://ubpred.org/) was used to predict potential ubiquitination sites in the N-terminal region of BRD7. **C.** Ubiquitination analysis was performed to detect the ubiquitination site of BRD7 in MCF7 cells co-transfected with Flag-TRIM25, the lysine mutant HA-BRD7, as well as and HA-Ub plasmids.

A JASPAR analysis results for YB1 binding sites located within the promoter of Bcl-2

Relative score	Start	End	Strand	Predicted sequence
0.87164176	1648	1656	+	CGCGCCACC
0.8710718	919	927	-	CCTAACACC
0.850419	782	790	-	CGCACTTTC

B

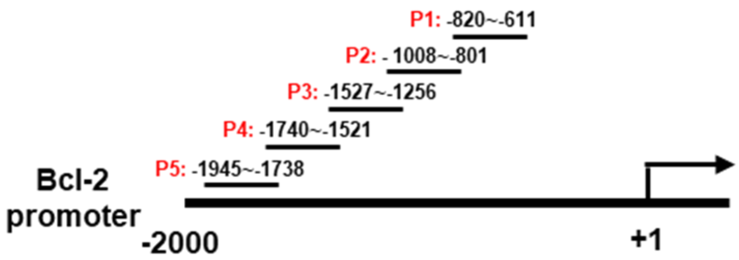

Figure. S5 YB1 directly target the promoter of Bcl-2. **A.** JASPAR analysis results for YB1 binding sites located within the promoter of Bcl-2 (JASPAR-A database of transcription factor binding profiles, relative score>0.85). **B.** P1, P2, P3, P4 and P5 were the primer positions for ChIP-qPCR.

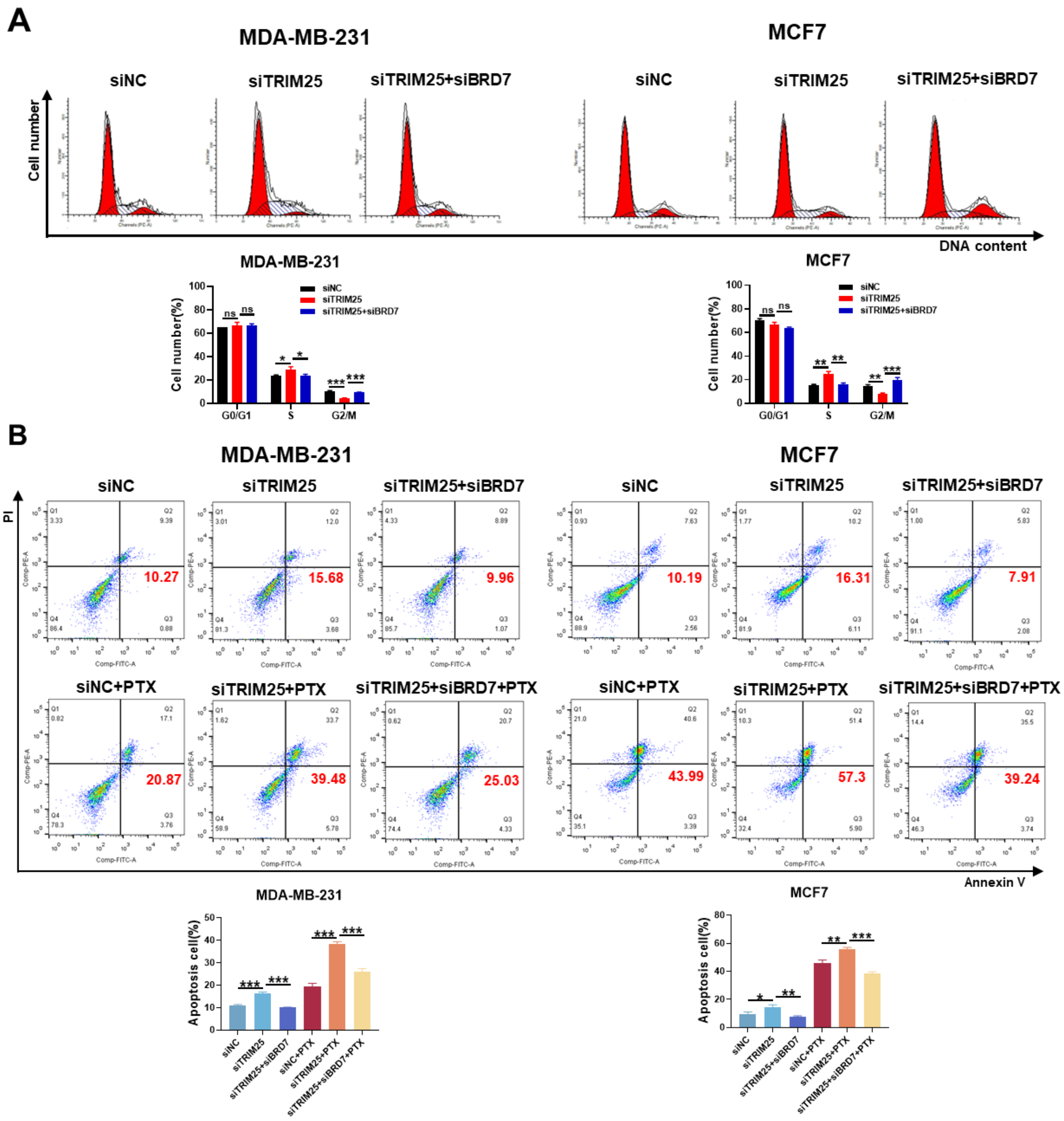

Figure. S6 TRIM25 knockdown partly inhibits cell cycle progression and promotes paclitaxel chemotherapy sensitivity through BRD7 degradation. **A.** The cell cycle process of the TRIM25 knockdown or BRD7 expression restored BC cells were further detected and analyzed. **B.** Flow cytometry showing the percentage of apoptotic cells in the TRIM25 knockdown and restoring BRD7 expression BC cells treated with or without PTX for 48 h. Data are shown as the mean ± SD of at least three independent experiments, and the significant level was identified by ns, no significance, *P < 0.05, **P< 0.01, and ***P < 0.001.

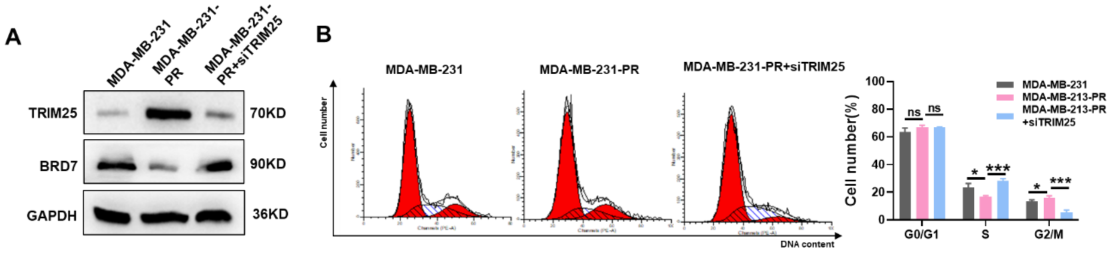

Figure. S7 TRIM25 knockdown reverses the cell cycle progression in PTX-resistant breast cancer cells. **A.** Western blot was performed to detect the expression of TRIM25 and BRD7 in each group. **B.** The cell cycle progression was further analyzed in each group. Data are shown as the mean ± SD of at least three independent experiments, and the significant level was identified by ns, no significance, *$P < 0.05$, and ***$P < 0.001$.

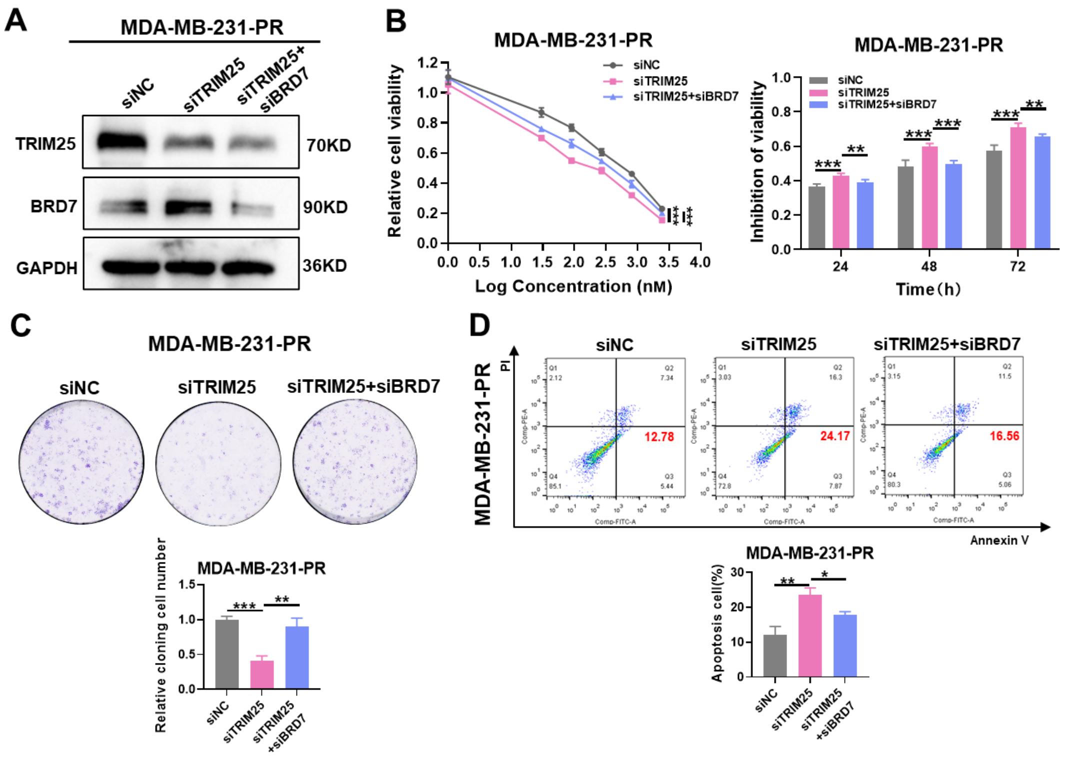

Figure. S8 TRIM25 knockdown increases chemosensitivity of paclitaxel partially through the degradation of BRD7 in PTX-resistant BC cells. **A.** Western blot was performed to detect the expression of TRIM25 and BRD7 in each group. **B.** CCK-8 assay was used to detect the cell viability (PTX: 0 nM, 1 nM, 30 nM, 90 nM, 270 nM, 810 nM, 2430 nM) and the cell inhibition rate (PTX: 200 nM) of TRIM25 knockdown or BRD7 expression restored MDA-MB-231-PR cells treated with PTX. **C.** The colony formation of PTX-resistant BC cells with TRIM25 knockout or BRD7 expression recovery was detected and analyzed. **D.** Flow cytometry showing the percentage of apoptotic cells in PTX-resistant BC cells treated with PTX for 48 hours, in which TRIM25 was knockdown and BRD7 expression was restored. PR, paclitaxel resistance. Data are shown as the mean ± SD of at least three independent experiments, and the significant level was identified by *$P < 0.05$, **$P < 0.01$, and ***$P < 0.001$.

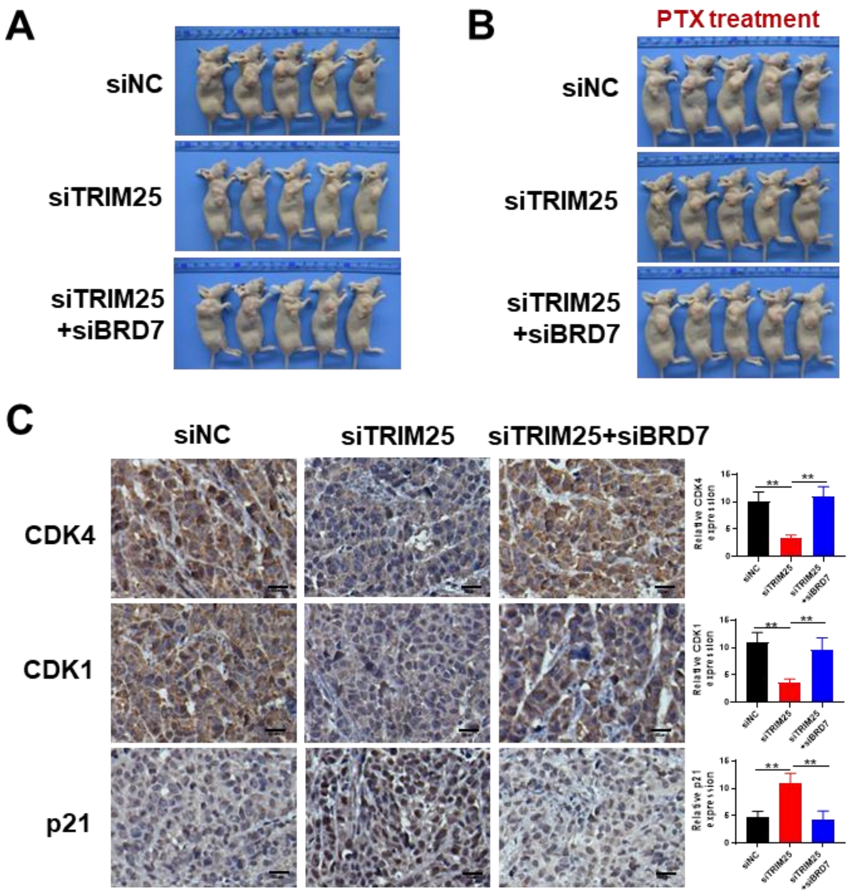

Figure. S9 TRIM25 promotes tumor growth and PTX resistance through degradation of BRD7 protein in vivo. **A.** Macroscopic mice of xenograft model, n = 5 per group. B. Macroscopic mice of xenograft model treated with PTX, n = 5 per group. **C.** CDK4, CDK1 and p21 expression in the tumor of nude mice were detected by IHC. Scale bar, 50 μm. Data are shown as the mean ± SD, and the significant level was identified by $^{**}P < 0.01$.
