# Supplementary material for: TRIM25 degrades BRD7 protein stability through the ubiquitin proteasome pathway to promote breast cancer progression and paclitaxel resistance by activating YB1/Bcl-2 transcription axis
Source: Cell Death Dis. 2025 Nov 28;16(1):872. doi: 10.1038/s41419-025-08140-8 (PMC12663559; doi:10.1038/s41419-025-08140-8)

Original blots and gels in this manuscript

1. Original blots used in Figure 1A

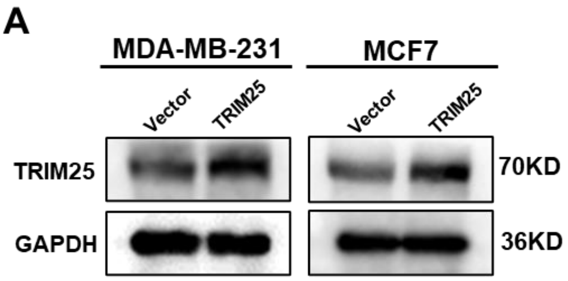

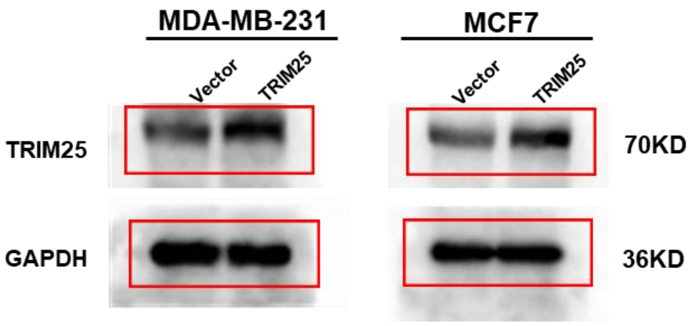

2. Original blots used in Figure 1B

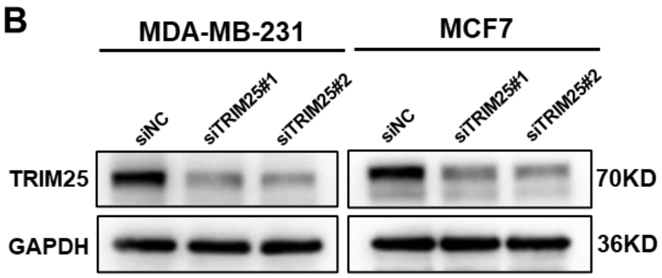

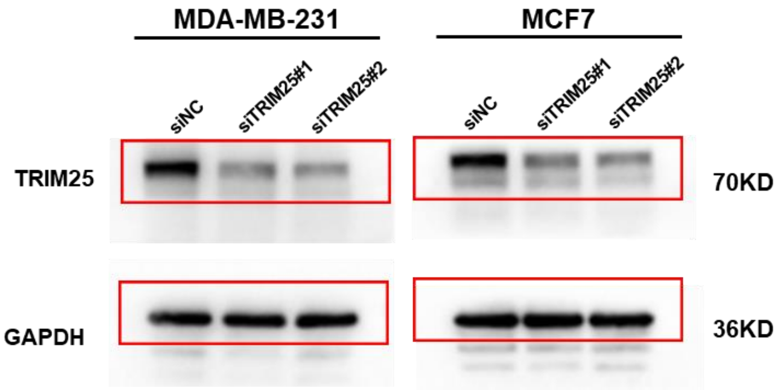

3. Original blots used in Figure 2E

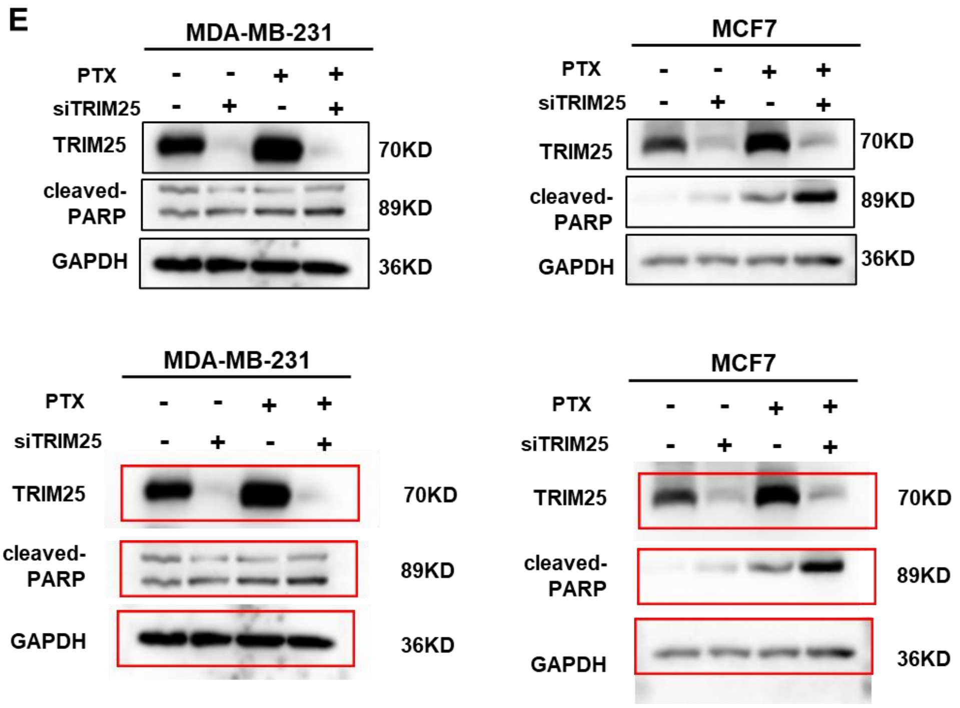

4. Original blots used in Figure 3A

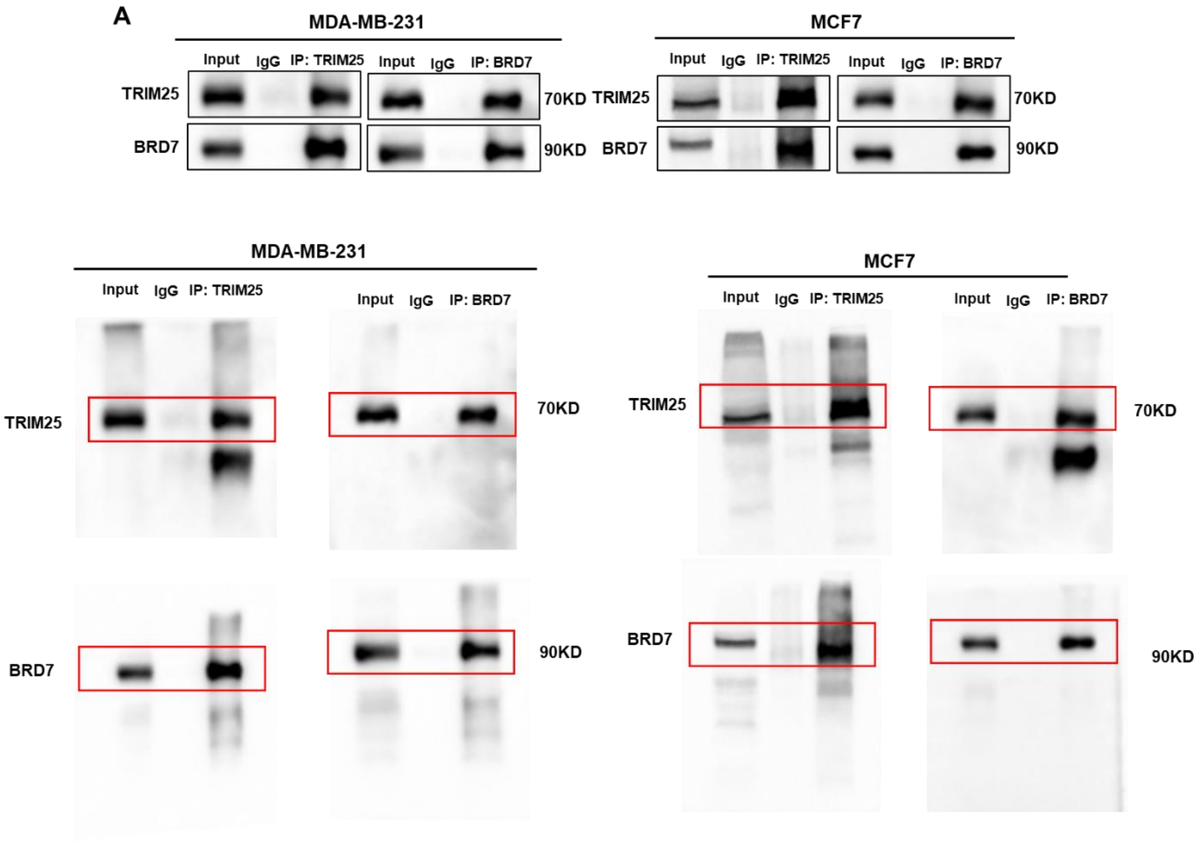

5. Original blots used in Figure 3E

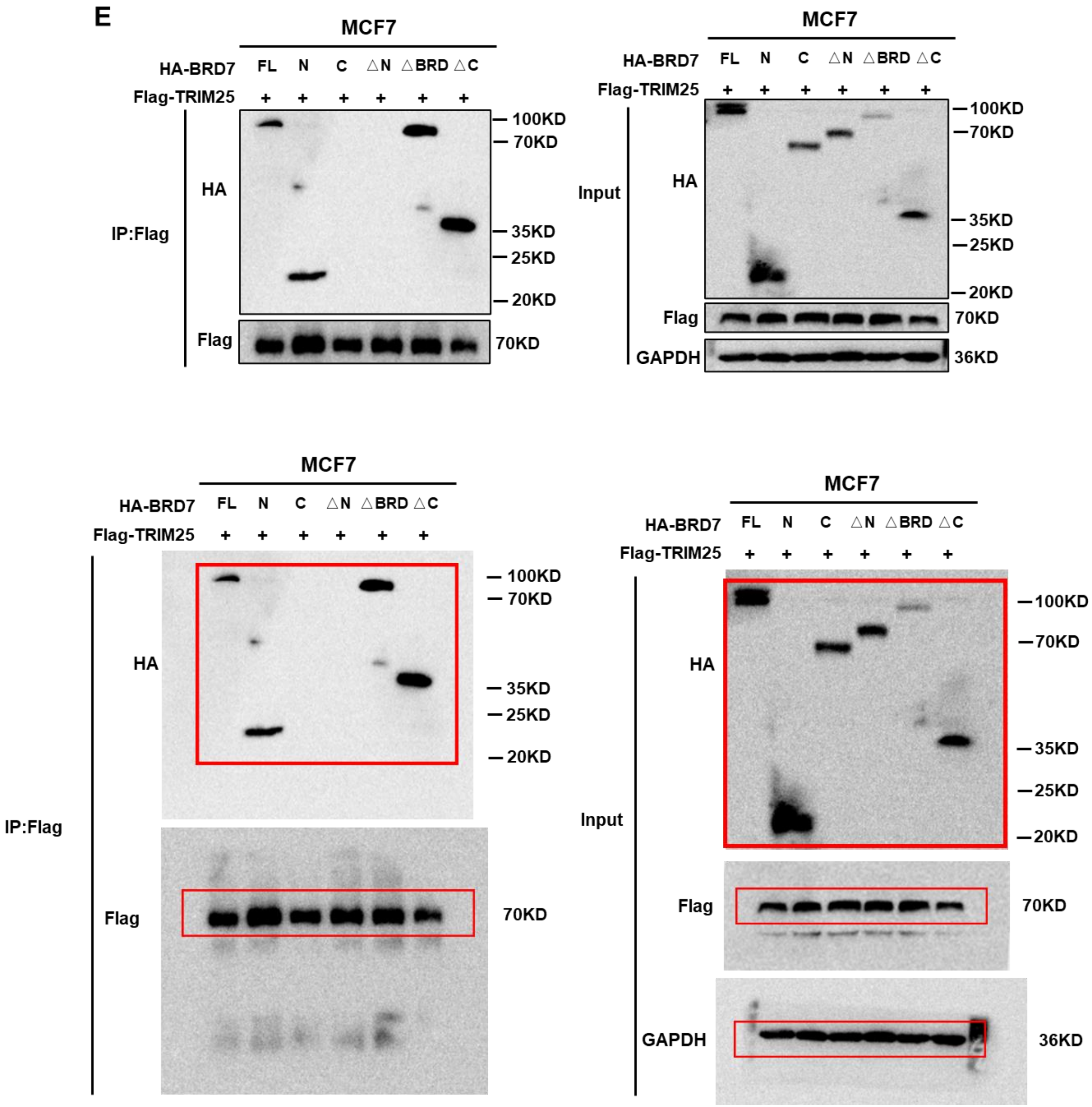

6. Original blots used in Figure 3F

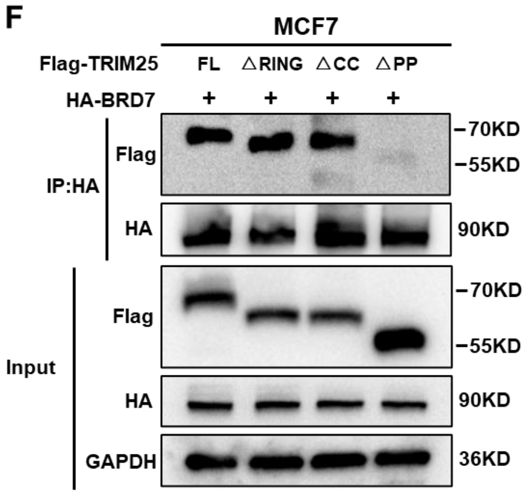

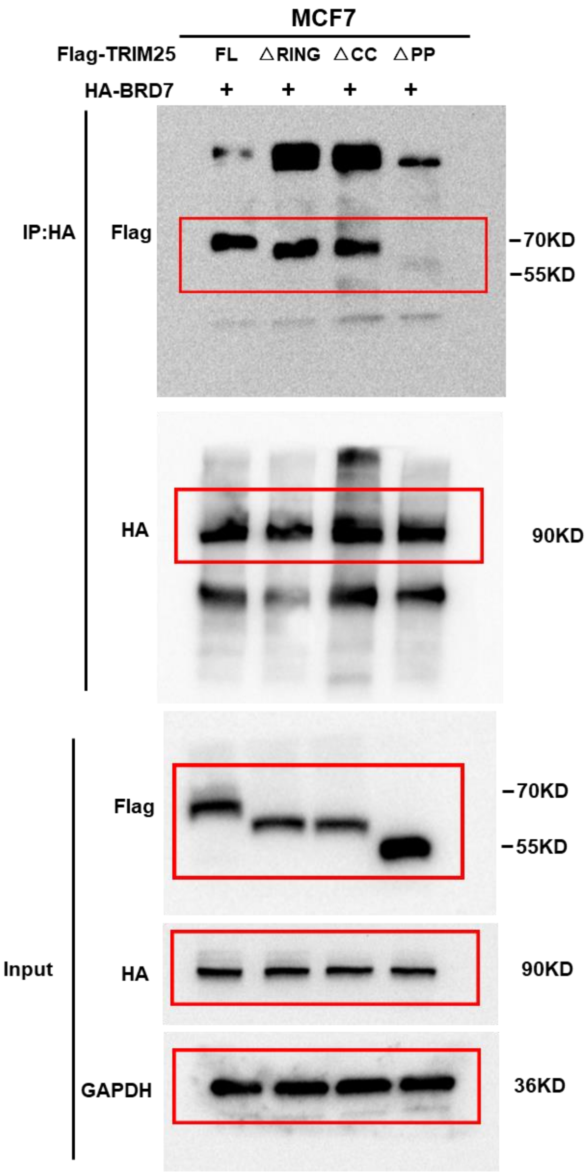

7. Original blots used in Figure 4A

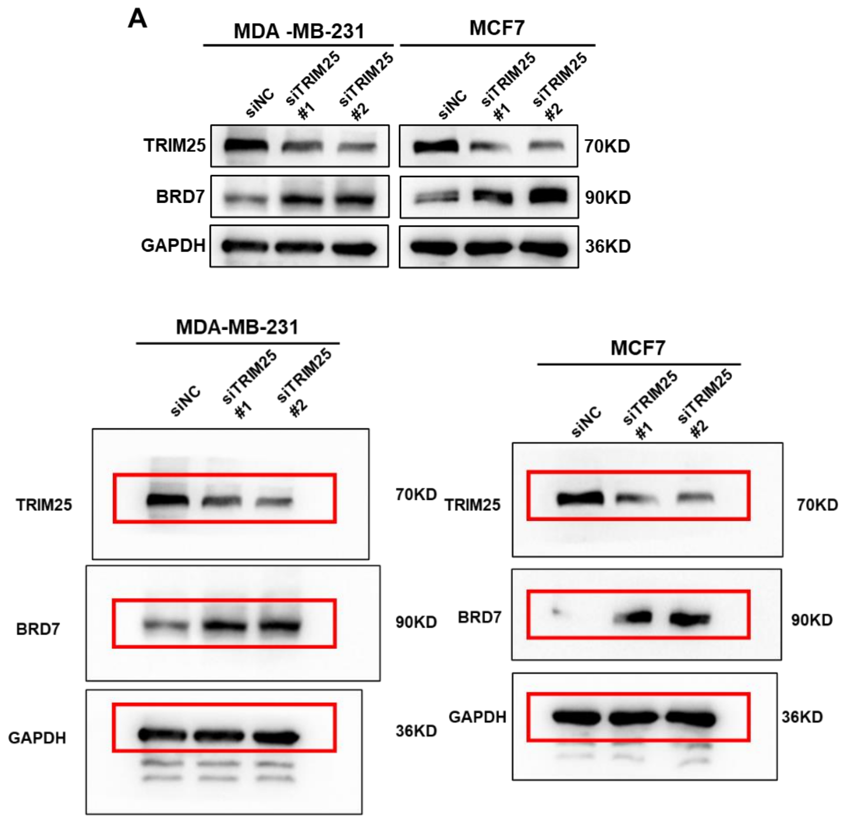

8. Original blots used in Figure 4B

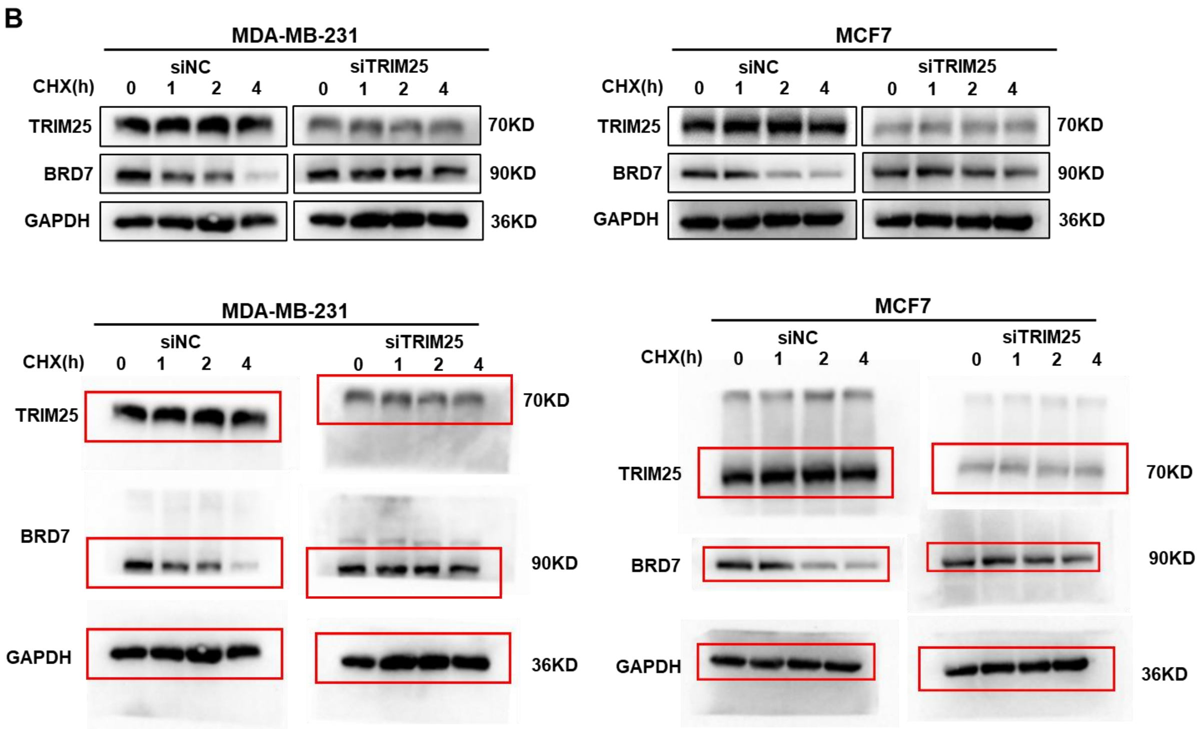

9. Original blots used in Figure 4C

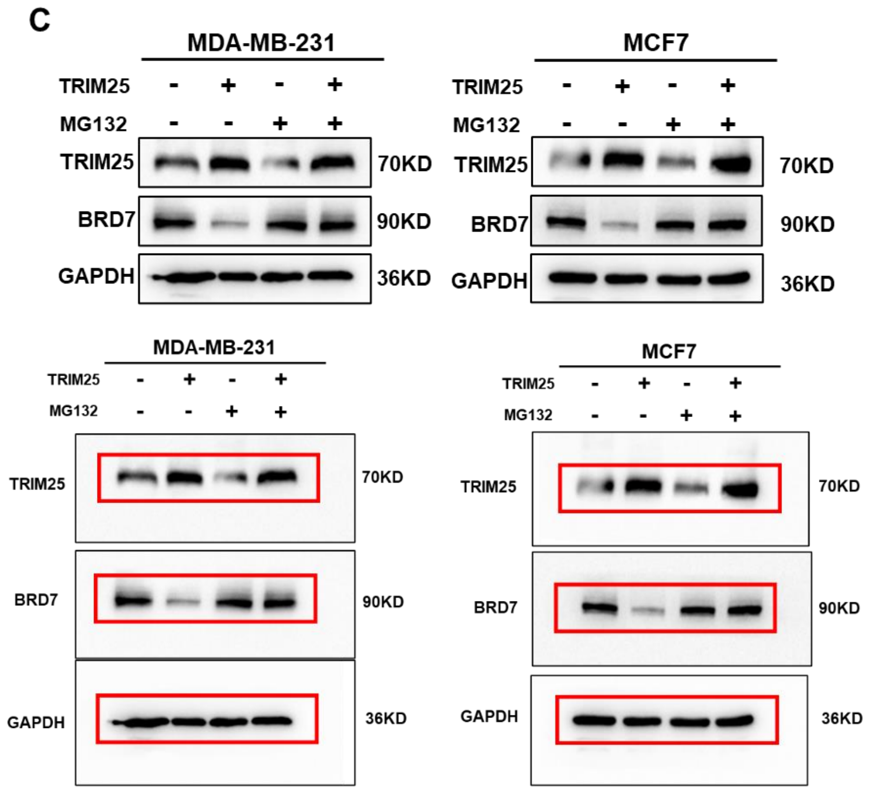

10. Original blots used in Figure 4D

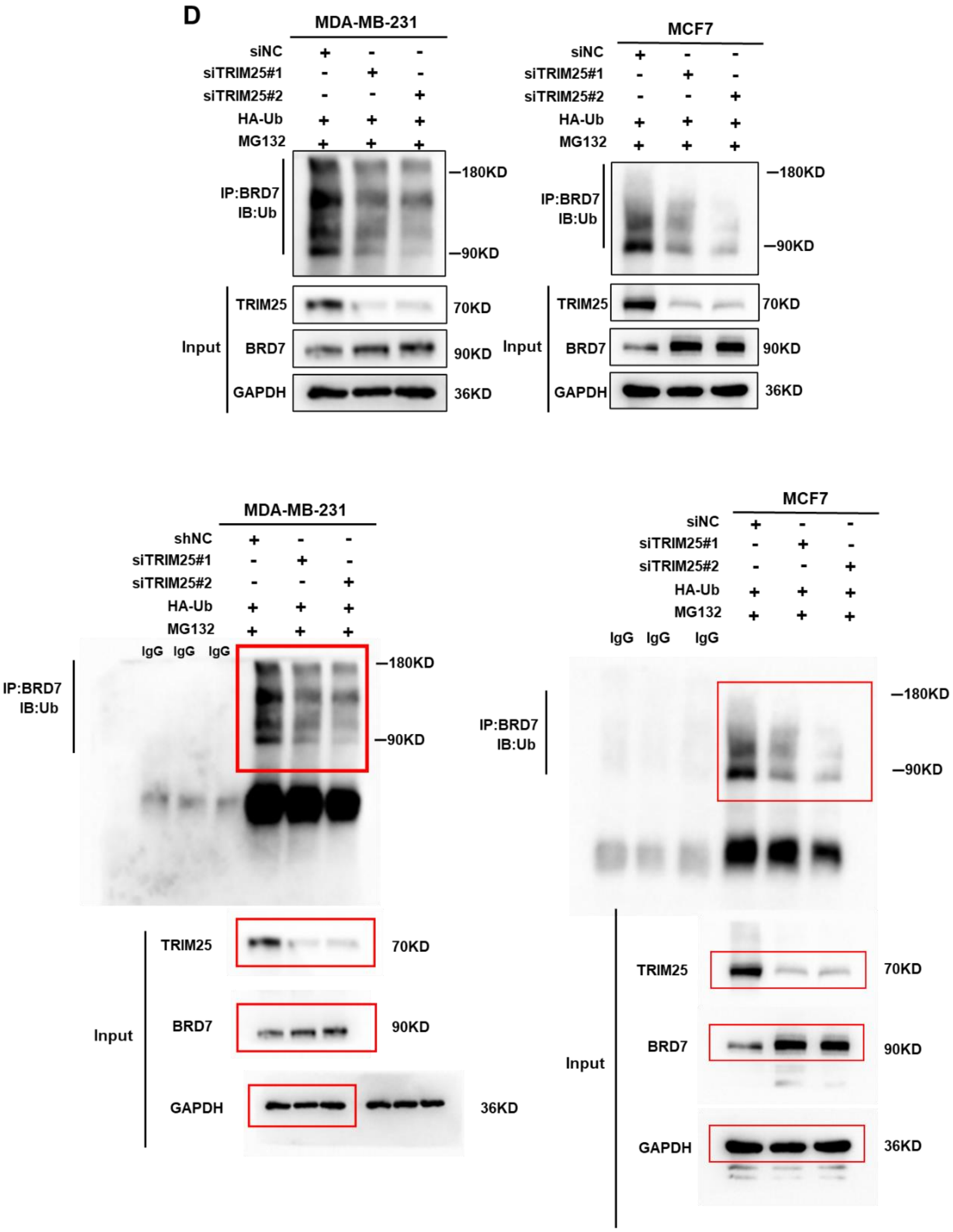

11. Original blots used in Figure 4E

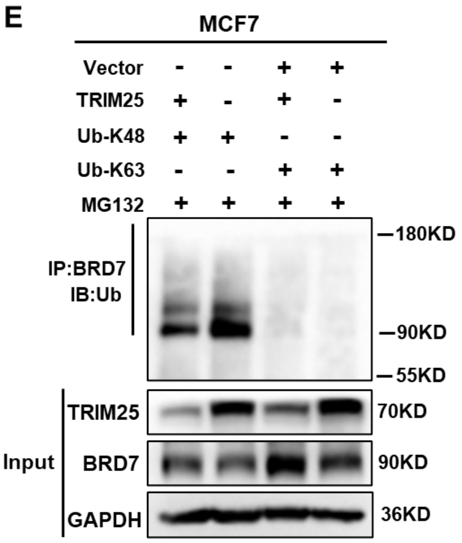

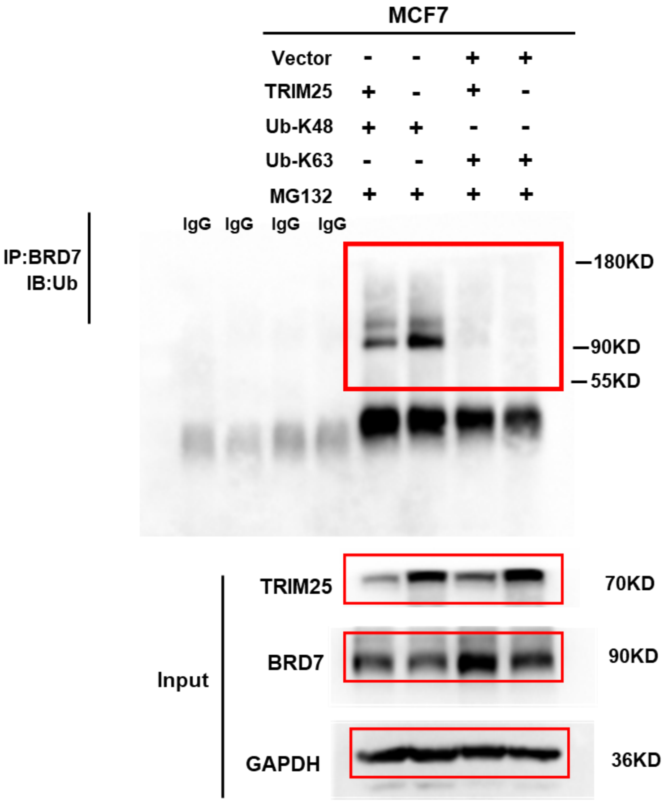

12. Original blots used in Figure 4F

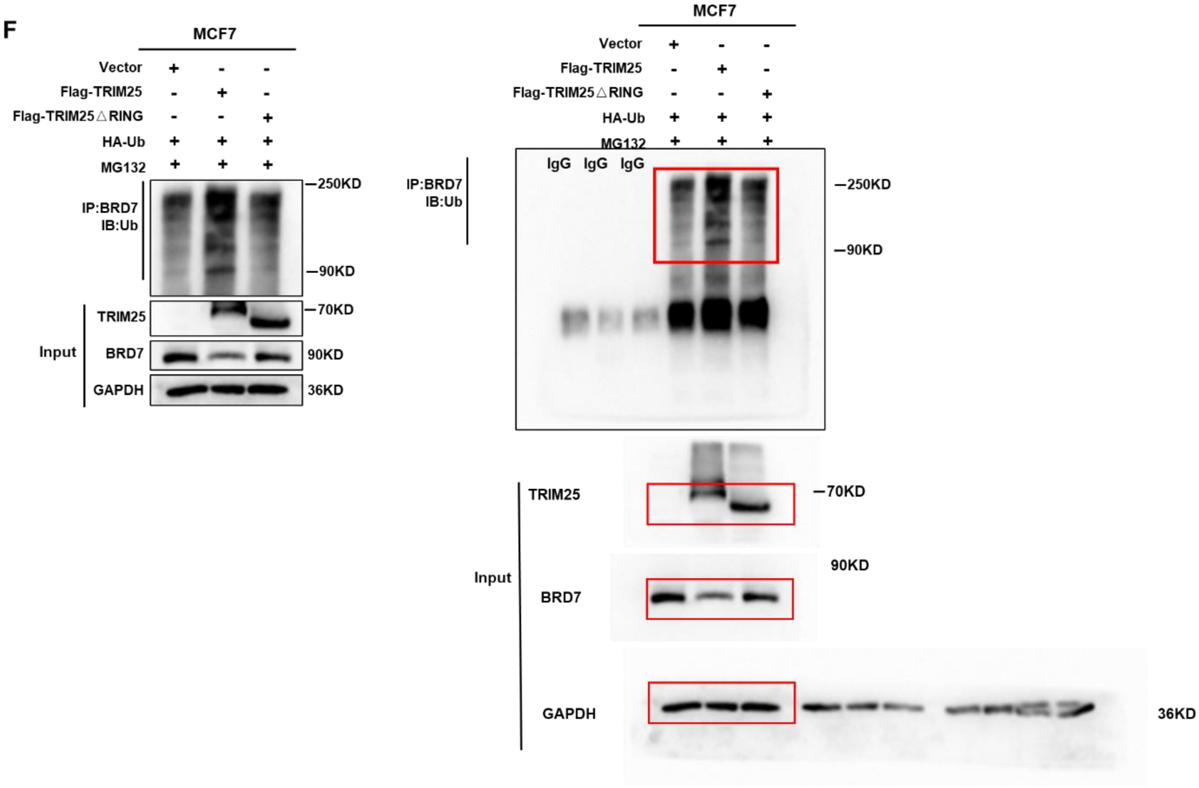

13. Original blots used in Figure 5A

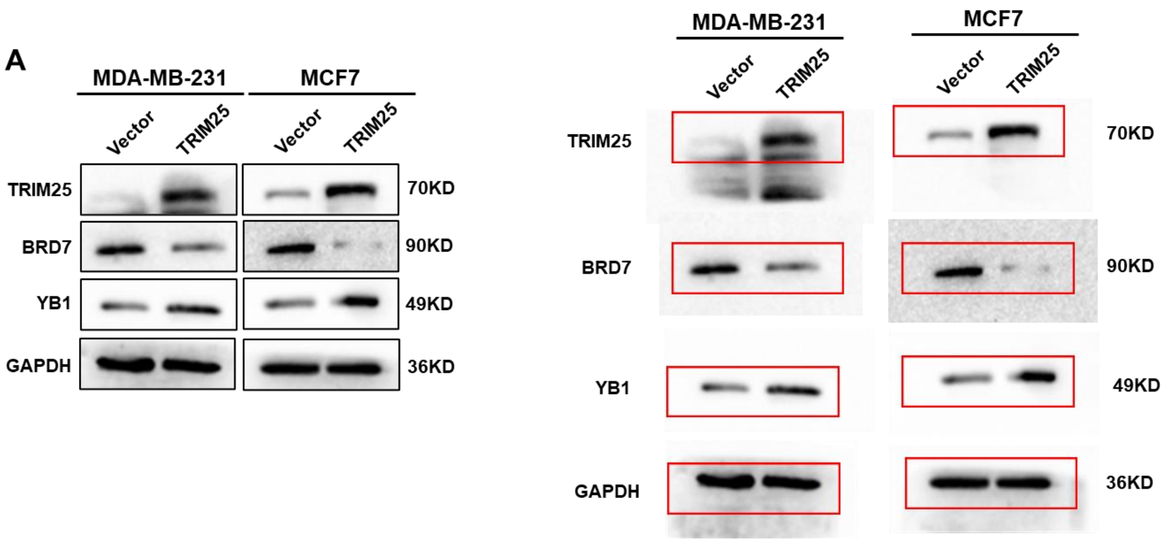

14. Original blots used in Figure 5B

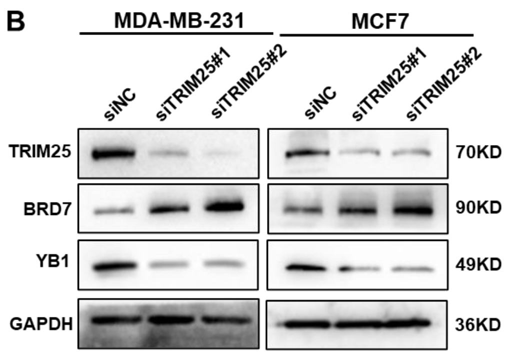

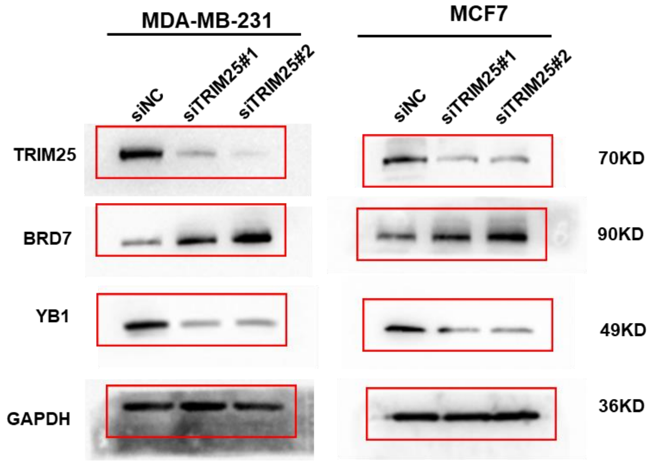

15. Original blots used in Figure 5D

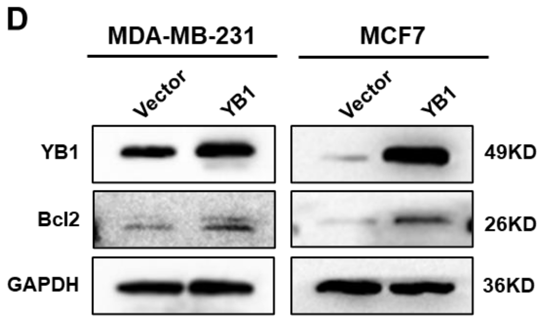

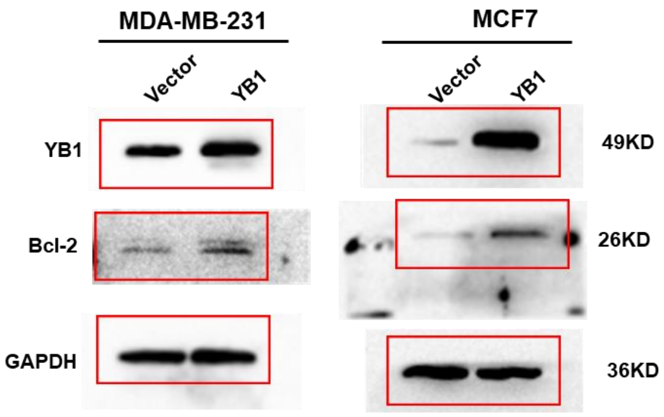

16. Original blots used in Figure 5E

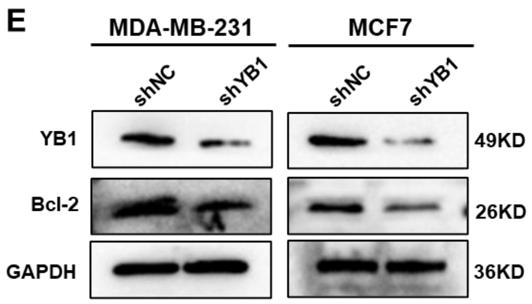

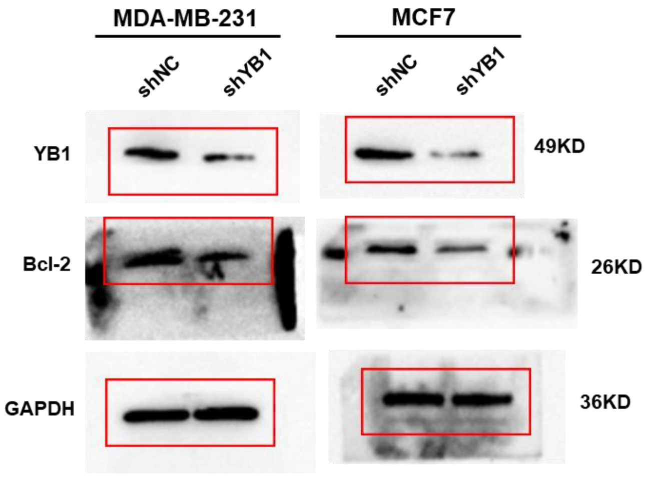

17. Original blots used in Figure 5G

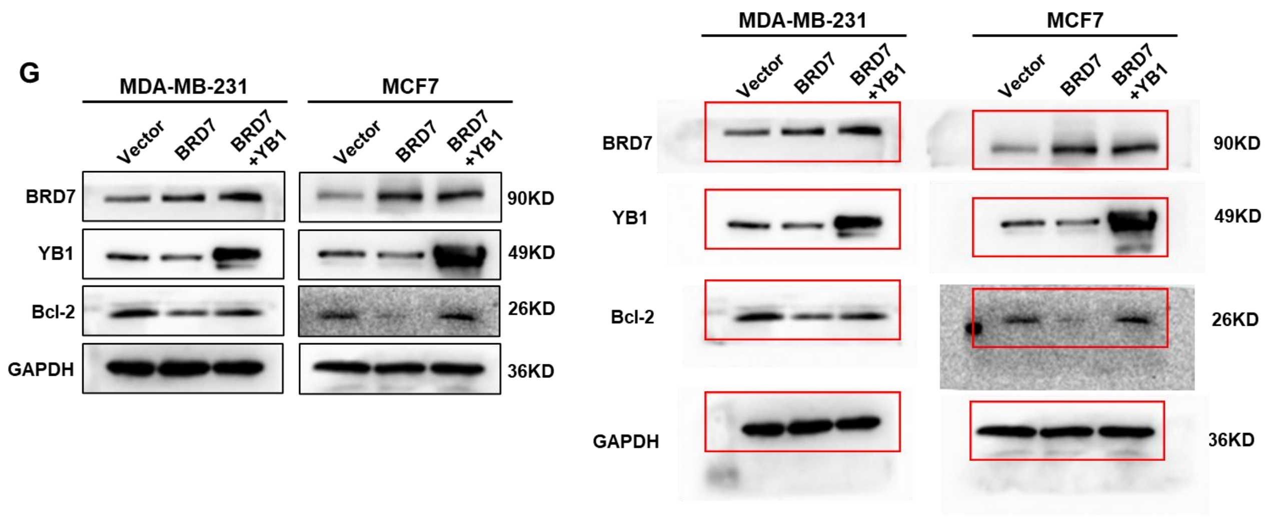

18. Original blots used in Figure 5H

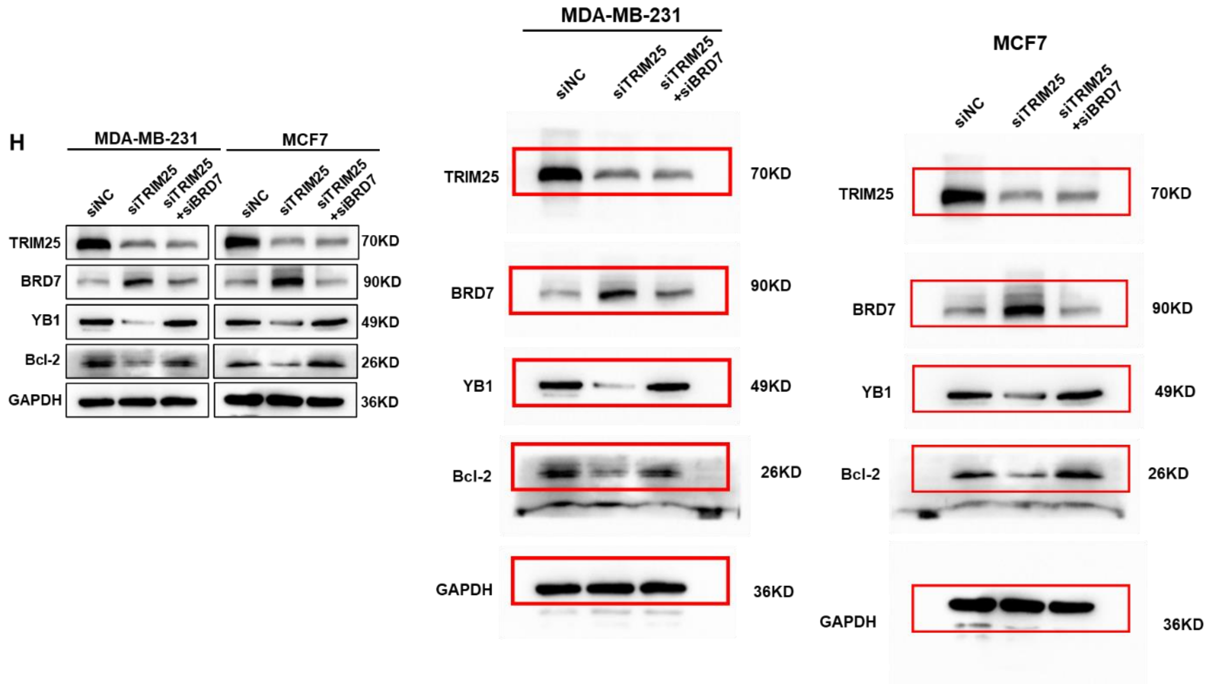

19. Original blots used in Figure 6A

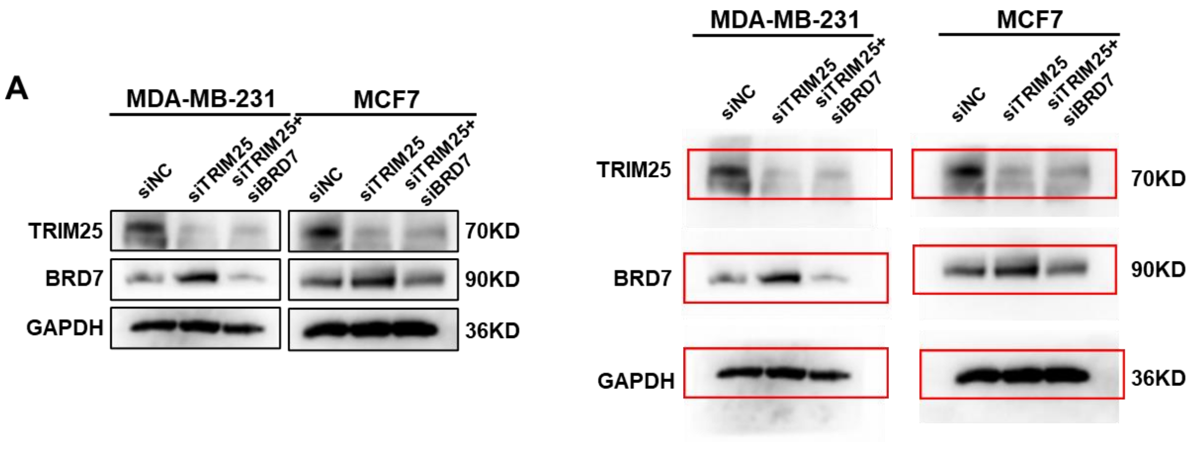

20. Original blots used in Figure 6D

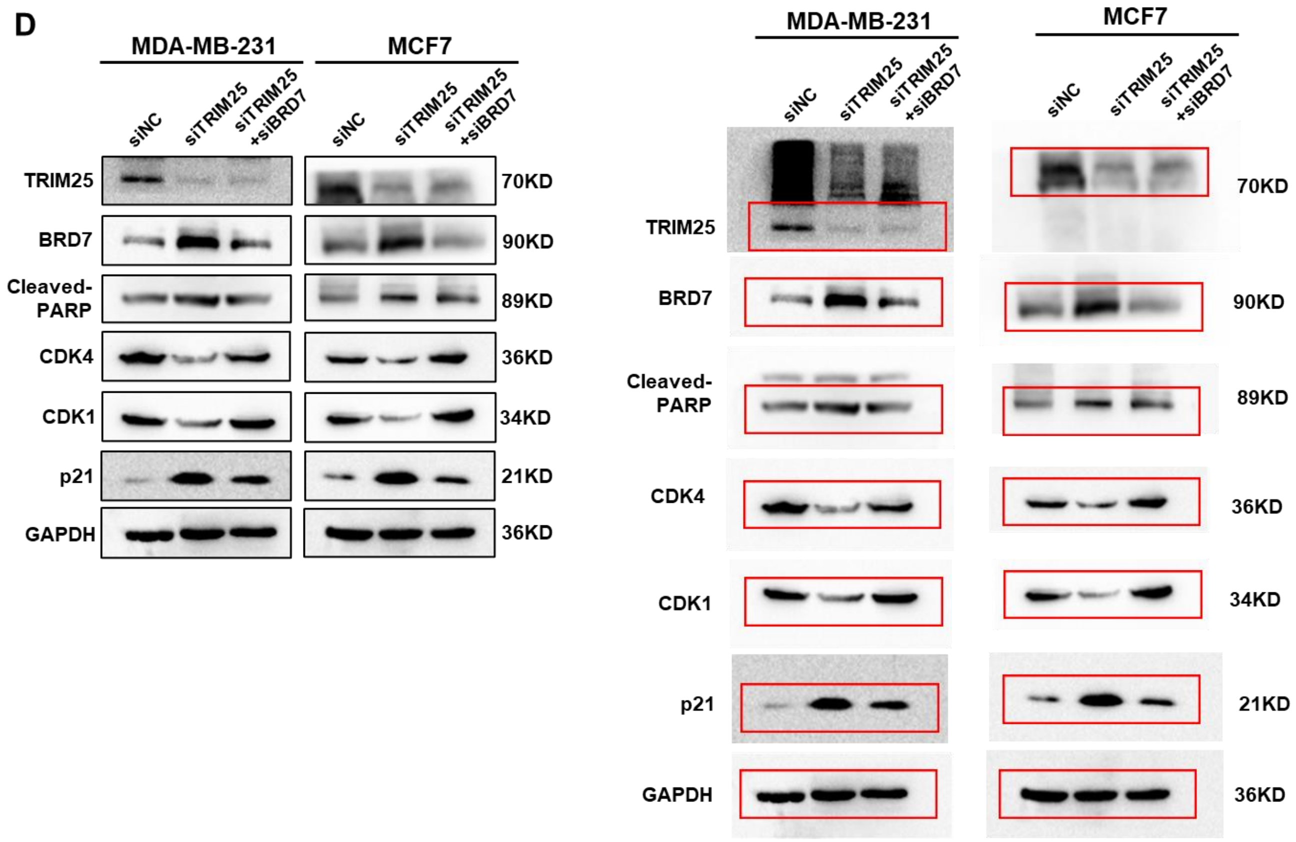

21. Original blots used in Figure S1E

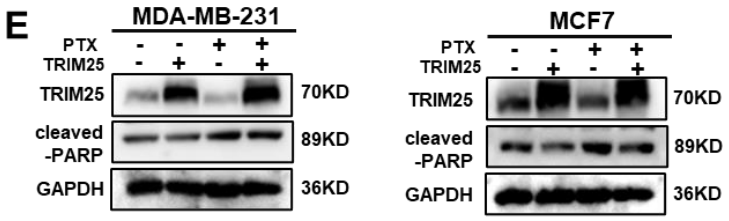

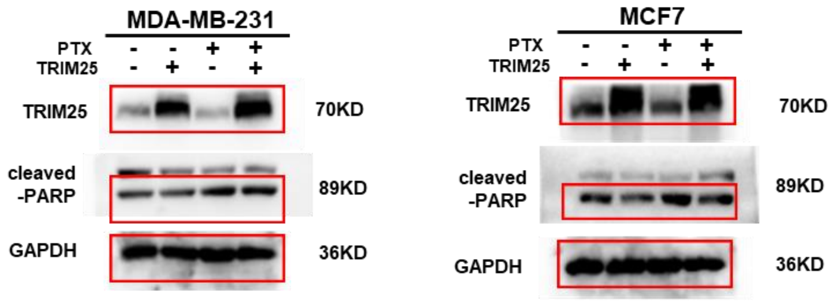

22. Original blots used in Figure S2B

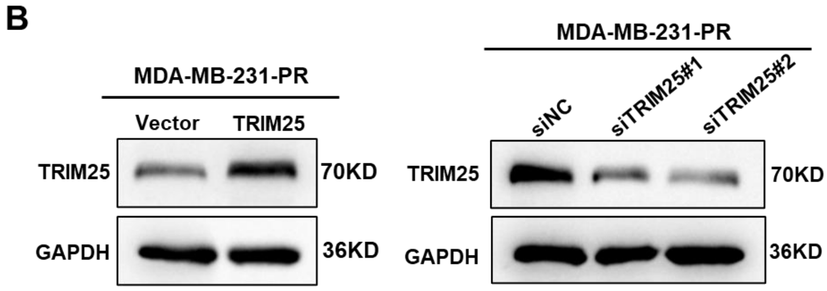

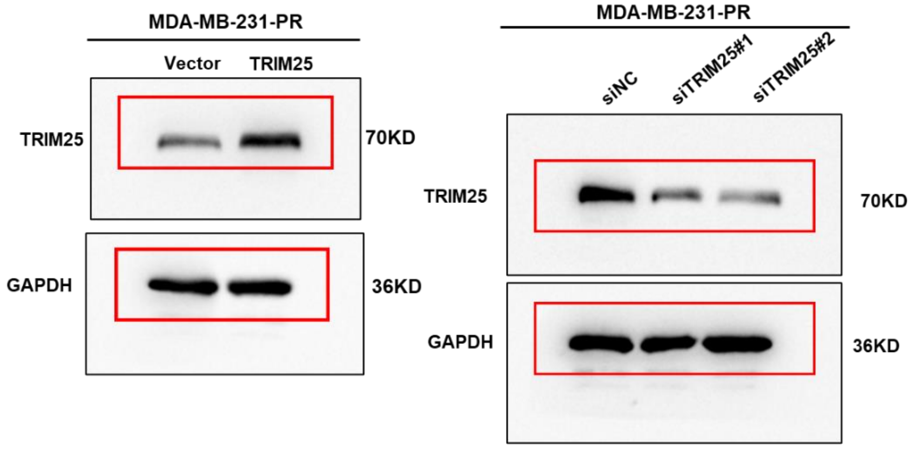

23. Original blots used in Figure S2G

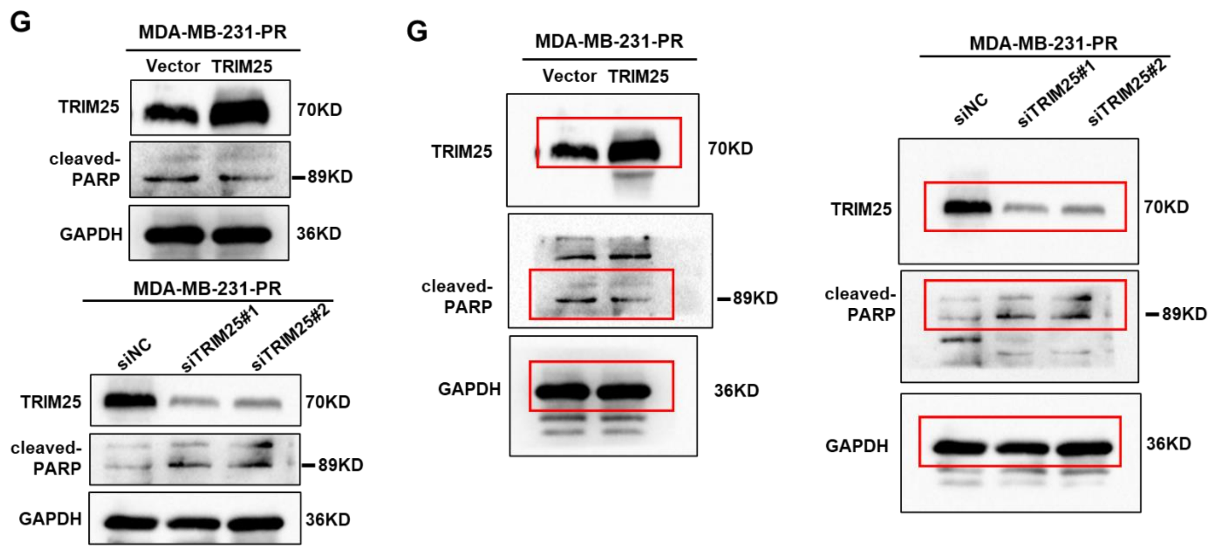

24. Original blots used in Figure S3C

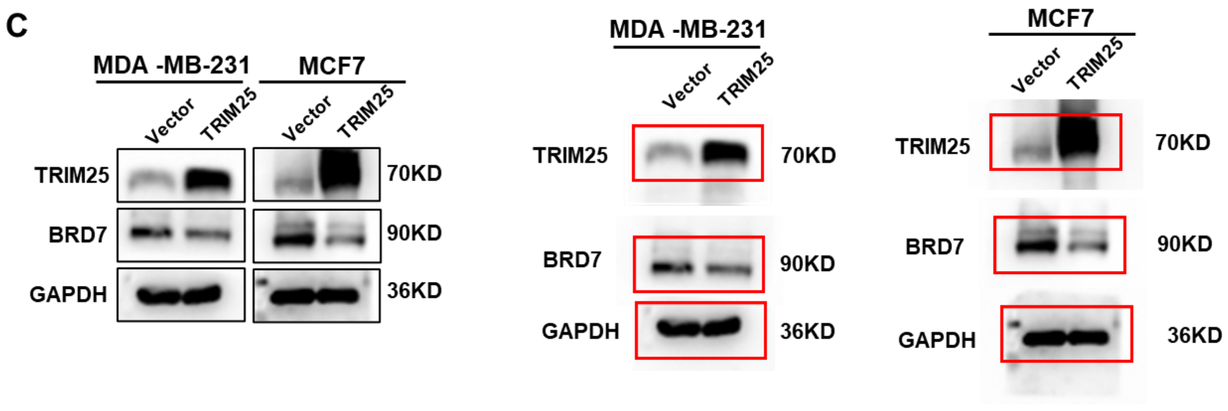

25. Original blots used in Figure S3D

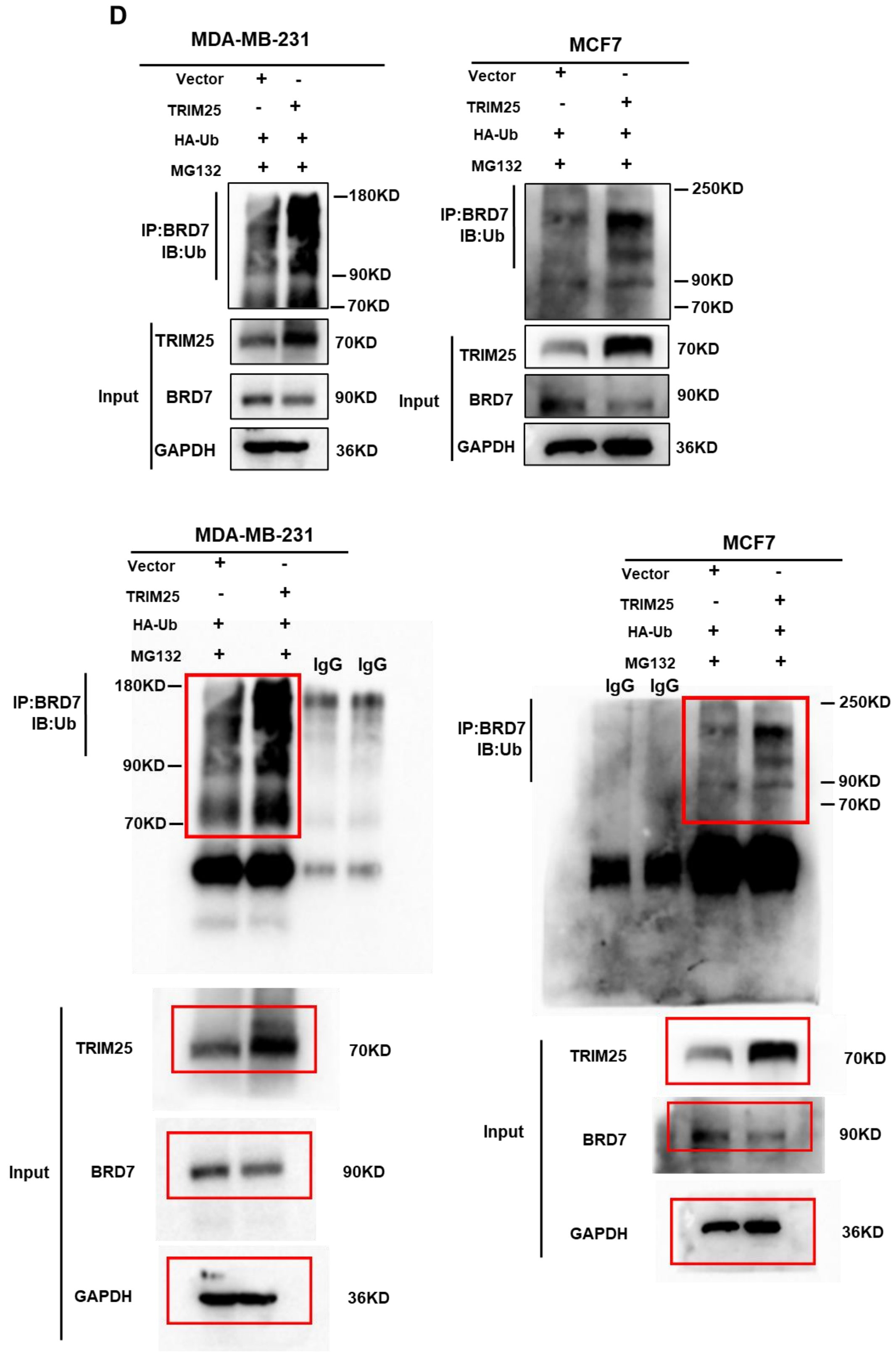

26. Original blots used in Figure S4A

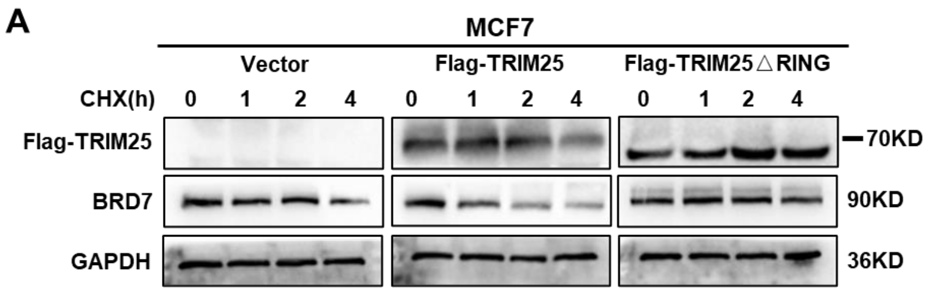

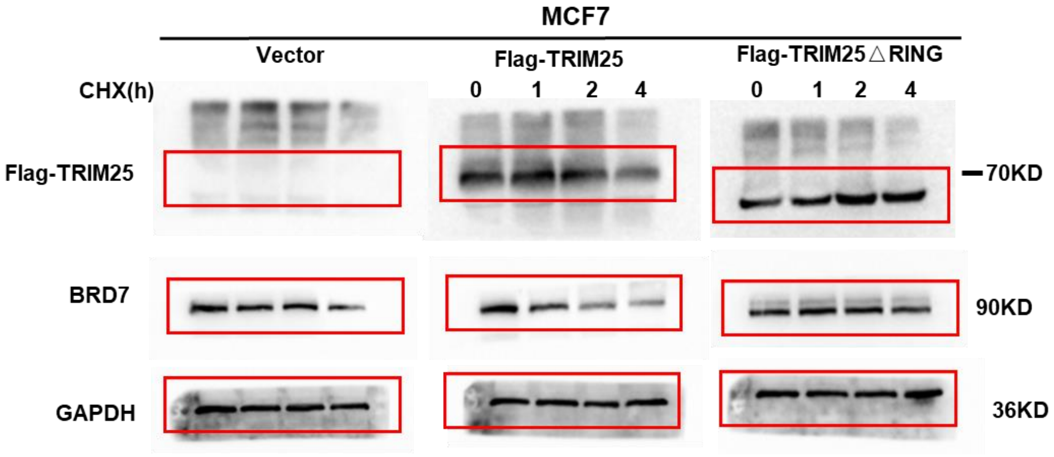

27. Original blots used in Figure S4C

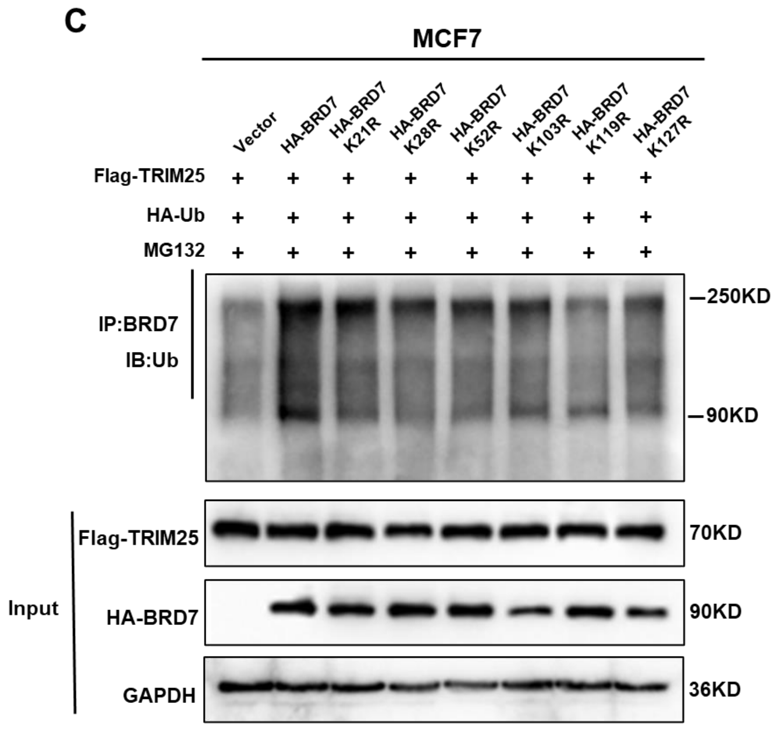

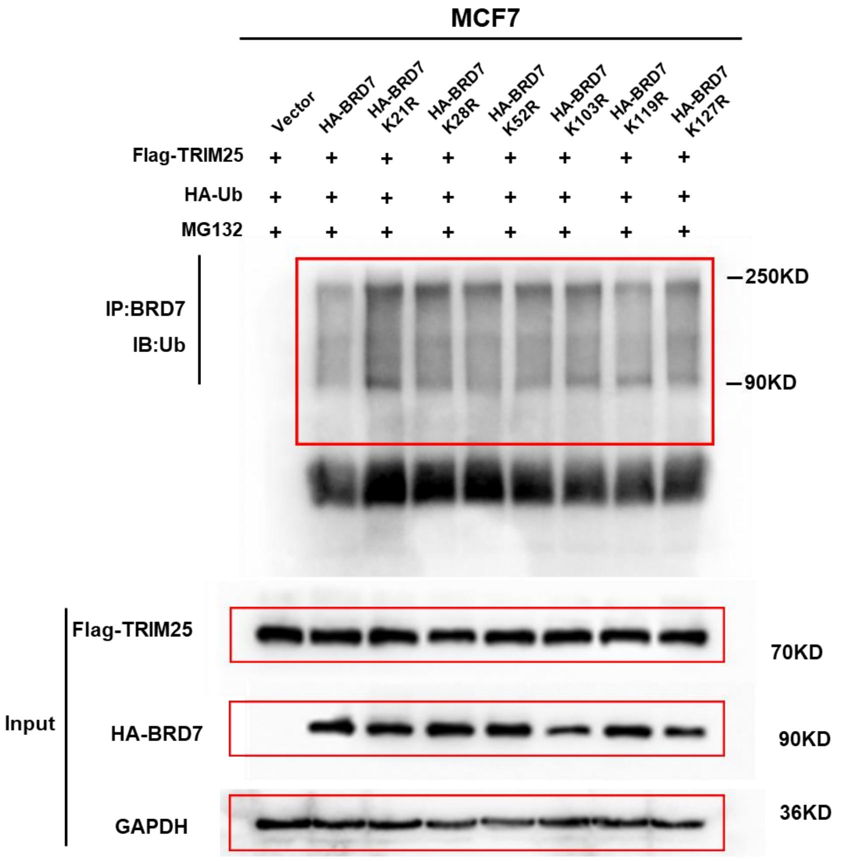

28. Original blots used in Figure S7A

A

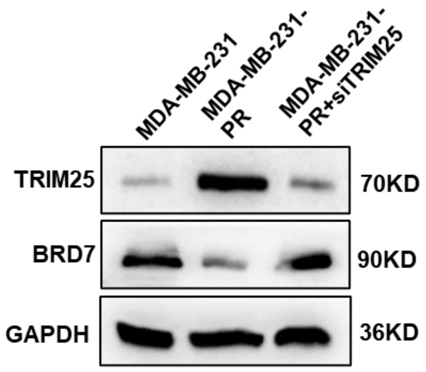

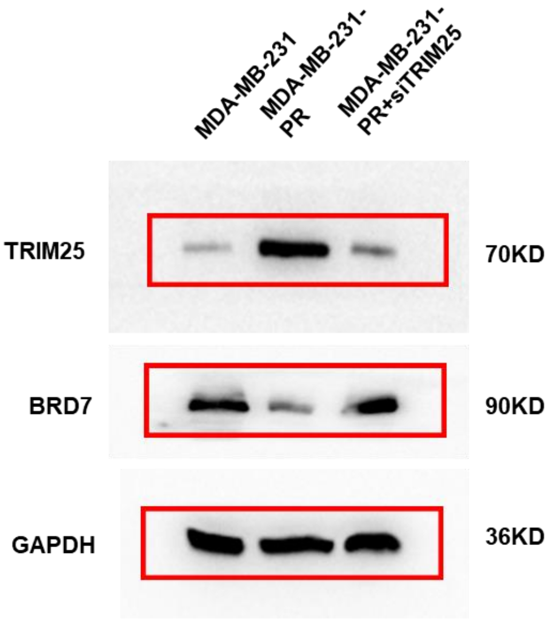

29. Original blots used in Figure S8A

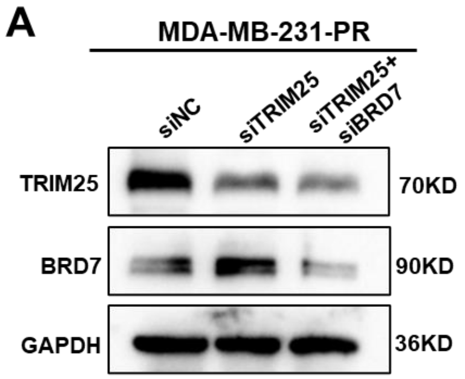

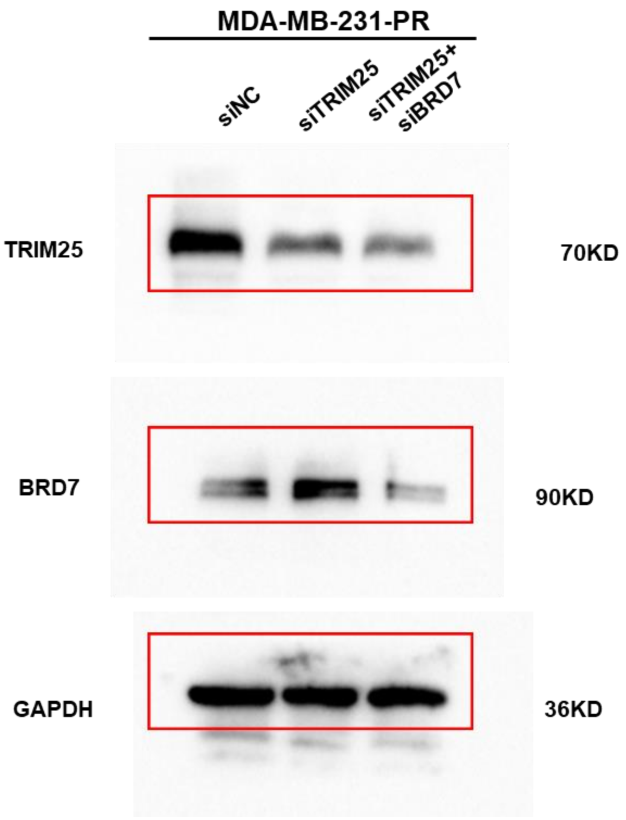

Supplement: Supplementary file 3 — Revised Original blots and gels in this manuscript [file 41419_2025_8140_MOESM3_ESM.pdf]
